# Potent and uniform fetal hemoglobin induction via base editing

**DOI:** 10.1038/s41588-023-01434-7

**Published:** 2023-07-03

**Authors:** Thiyagaraj Mayuranathan, Gregory A. Newby, Ruopeng Feng, Yu Yao, Kalin D. Mayberry, Cicera R. Lazzarotto, Yichao Li, Rachel M. Levine, Nikitha Nimmagadda, Erin A. D. Dempsey, Guolian Kang, Shaina N. Porter, Phillip A. Doerfler, Jingjing Zhang, Yoonjeong Jang, Jingjing Chen, Henry W. Bell, Merlin Crossley, Senthil Velan Bhoopalan, Akshay Sharma, John F. Tisdale, Shondra M. Pruett-Miller, Yong Cheng, Shengdar Q. Tsai, David R. Liu, Mitchell J. Weiss, Jonathan S. Yen

**Affiliations:** 1 Department of Hematology, St. Jude Children’s Research Hospital, Memphis, TN, USA.; 2 Merkin Institute of Transformative Technologies in Healthcare, Broad Institute of Harvard and MIT, Cambridge, MA, USA.; 3 Department of Chemistry and Chemical Biology, Harvard University, Cambridge, MA, USA.; 4 Howard Hughes Medical Institute, Harvard University, Cambridge, MA, USA.; 5 Department of Biostatistics, St. Jude Children’s Research Hospital, Memphis, TN, USA.; 6 Department of Cell and Molecular Biology, St. Jude Children’s Research Hospital, Memphis, TN, USA.; 7 School of Biotechnology and Biomolecular Sciences, University of New South Wales, Sydney, New South Wales, Australia; 8 Department of Bone Marrow Transplantation and Cellular Therapy, St. Jude Children’s Research Hospital, Memphis, TN, USA.; 9 Cellular and Molecular Therapeutics Branch, National Heart, Lung, and Blood Institute and National Institute of Diabetes and Digestive and Kidney Diseases, Bethesda, MD, USA.; 10 These authors contributed equally: Thiyagaraj Mayuranathan, Gregory A. Newby.

## Abstract

Inducing fetal hemoglobin (HbF) in red blood cells (RBCs) can alleviate β-thalassemia and sickle cell disease. We compared five strategies in CD34^+^ hematopoietic stem and progenitor cells (HSPCs), using either Cas9 nuclease or adenine base editors (ABE). The most potent modification was ABE generation of γ-globin −175 A>G; homozygous edited erythroid colonies expressed 81±7% HbF versus 17±11% in unedited controls, whereas HbF levels were lower and more variable for two Cas9 strategies targeting a BCL11A binding motif in the γ-globin promoter or a *BCL11A* erythroid enhancer. The −175 A>G base edit also induced HbF more potently than a Cas9 approach in RBCs generated after transplantation of CD34^+^ HSPCs into mice. Our data suggest a strategy for potent, uniform induction of HbF and provide insights into γ-globin gene regulation. More generally, we demonstrate that diverse indels generated by Cas9 can cause unexpected phenotypic variation that can be circumvented by base editing.

## Introduction

Sickle cell disease (SCD) and β-thalassemia are caused by mutations in the *HBB* gene, which encodes the β-globin subunit of adult hemoglobin (HbA, α2β2)^1,2^. These disorders become symptomatic after birth, following the switch in expression from fetal γ-globin genes (*HBG1/HBG2*) to mutated β-globin (*HBB*). Reversing this developmental switch through genetic modification of autologous hematopoietic stem cells (HSCs) represents a promising therapeutic approach^3^. Early clinical studies have demonstrated that suppressing the γ-globin repressor gene *BCL11A* by Cas9 nuclease–mediated disruption of its erythroid enhancer or RNA interference induces fetal hemoglobin (HbF, α2γ2) and reduces morbidities of SCD or β-thalassemia^4,5^.

Therapeutic reactivation of γ-globin expression is informed by hereditary persistence of fetal hemoglobin (HPFH), a benign genetic condition that alleviates the pathologies of co-inherited β-haemoglobinopathies^6^. Some forms of HPFH are caused by single-nucleotide variants or small deletions that eliminate γ-globin promoter binding motifs for the transcriptional repressors BCL11A or ZBTB7A^7^. Genome-editing nuclease disruption of these motifs via non-homologous end joining (NHEJ) in hematopoietic stem and progenitor cells (HSPCs) raises HbF expression to potentially therapeutic levels in RBC progeny^8–11^. Other γ-globin promoter variants cause HPFH by creating new DNA binding motifs for transcriptional activators^7^. These substitutions cannot be installed easily by nuclease editors because NHEJ, the default pathway for repair of double-stranded DNA breaks (DSBs) in HSCs, generates uncontrolled mixtures of insertion–deletion mutations (indels). Base editors, consisting of a nuclease-deficient Cas9 fused to a deaminase, install precise base-pair transitions^12,13^ and have been used to induce HbF by disrupting the +58 *BCL11A* erythroid enhancer or repressor binding motifs in the γ-globin promoter, in a similar manner to Cas9 nucleases^14,15^. Additionally, base editors have been used to induce HbF by creating new transcription factor binding sites in the γ-globin promoters^13,16–21^. The results of these studies indicate that base editing approaches can outperform Cas9-induced NHEJ strategies for HbF induction. This hypothesis is now being tested in a clinical trial (NCT05456880). However, the best genome-editing strategies for therapeutic HbF induction have yet to be identified.

Base editors have several potential advantages over Cas9. They can create precise base-pair changes efficiently without the requirement for homology-directed repair, which is difficult to achieve in HSCs without generating deleterious collateral indels. Additionally, base editing does not depend on intermediate DSBs that can cause TP53-mediated DNA damage responses and chromosomal abnormalities^22–25^. Base editors may be particularly useful for modifying the duplicated, tandemly arranged γ-globin genes *HBG1* and *HBG2*, because simultaneous Cas9 nuclease–induced DSBs at both loci can cause a 4.9-kb deletion of intervening DNA^8,26,27^.

To optimize genome editing for HbF induction, we used a therapeutically relevant strategy to compare five different approaches, including Cas9 disruption of the +58 *BCL11A* erythroid enhancer or of the γ-globin promoter BCL11A binding motif; or adenine base editor (ABE) installation of three γ-globin promoter HPFH variants located −198, −175, and −113 base pairs upstream of the transcriptional start site. Compared with Cas9-generated indels, base editing approaches were more potent, with −175 A>G causing the strongest induction of HbF by creating a new TAL1 binding motif that stimulates long-range interaction with the locus control region (LCR), a powerful upstream enhancer. We achieved clinically-relevant levels of −175 A>G editing in mouse-repopulating human HSCs, with significant reductions in hypoxia-induced sickling of SCD donor-derived RBCs. Base editing generated precise nucleotide changes with uniform HbF induction, whereas Cas9 generated indel mixtures with surprisingly variable %HbF induction even in cells with disrupted γ-globin promoter BCL11A binding motifs. Therefore, base editing represents a potentially superior alternative to Cas9 for therapeutic induction of HbF, partly by averting the generation of indel mixtures with varying biological consequences.

## Results

### Base editing to induce HbF in CD34^+^ cells.

**Extended Data Fig. 1 |:**
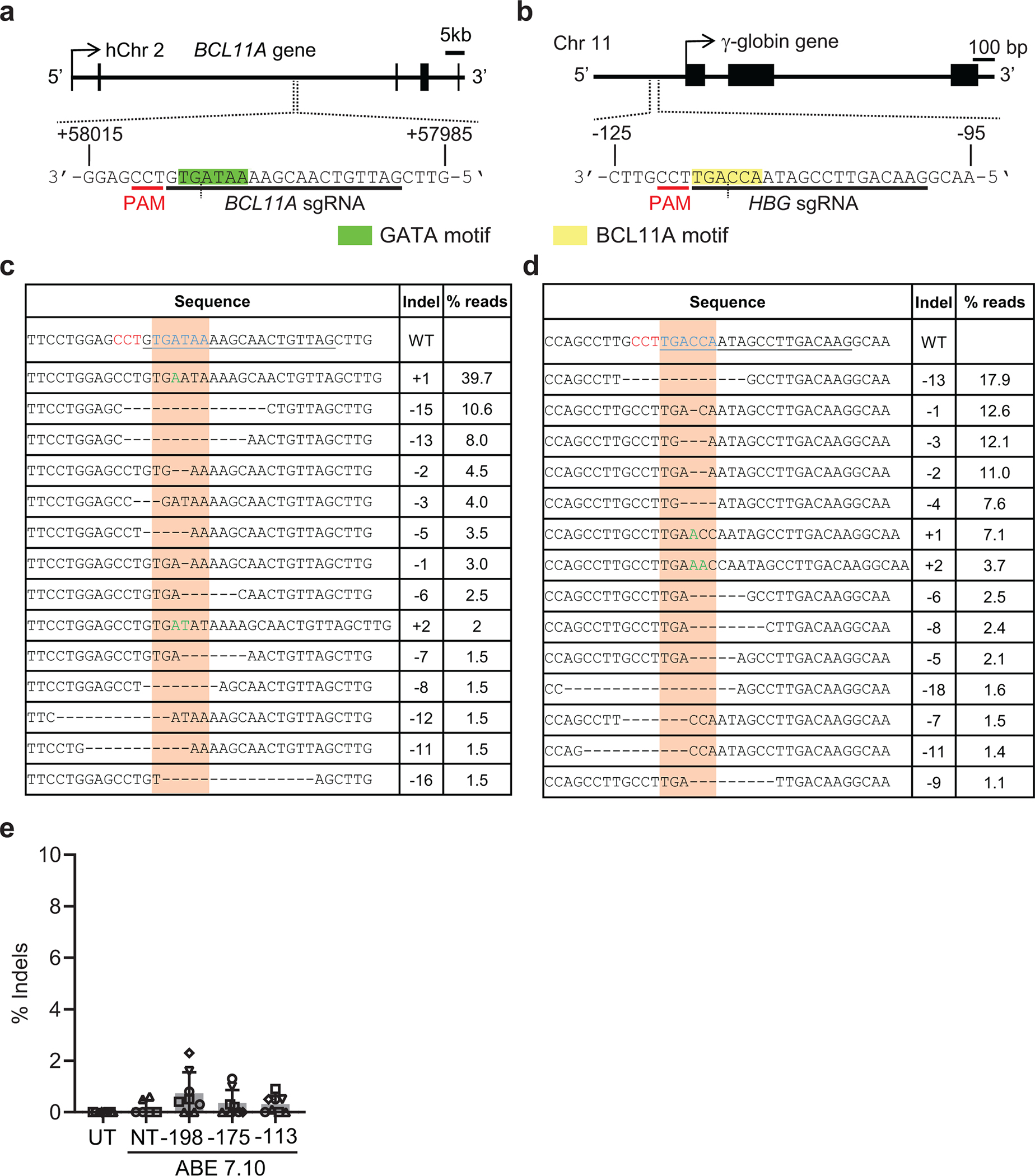
Insertion/deletion (indel) mutations generated in healthy donor CD34^+^ hematopoietic stem and progenitor cells (HSPCs) by Cas9 nuclease. **a**, *BCL11A* gene (hg19-chr2:60,722,392–60,722,422) showing the target GATA1 binding motif (TGATAA) in the +58 *BCL11A* erythroid enhancer highlighted in green. The single guide RNA (sgRNA) protospacer and protospacer-adjacent motifs (PAM) are shown in black and red, respectively. The vertical dotted line indicates the Cas9 cleavage site. **b**, The γ-globin (*HBG1/2*) gene (*HBG2*- hg19-chr11:5,276,106–5,276,136; *HBG1*- hg19-chr11:5,271,182–5,271,212) showing the target *BCL11A* binding motif (TGACCA) in the promoter highlighted in yellow. **c,d**, Sequence alignments of the *BCL11A* and γ-globin genes showing the most common Cas9 indels determined by next generation sequencing (NGS) 3 days after editing. The sgRNA sequences are underlined with PAM in red text. Targeted transcription factor binding motifs are indicated by the orange columns and shown in the wild type (WT) sequences as underlined blue text. Deletions are represented by dashes and insertions by green text. The % of each indel relative to total NGS reads is shown at right. **e**, Percentage of indels after base editing (n=8 for UT, −198, −175 and −113, n=6 for NT). Bar graphs show mean ± standard deviation (SD). Each symbol represents an independent experiment with different shapes representing unique HSPC donors. UT, untreated. NT, non-targeting gRNA.

**Fig. 1 |:**
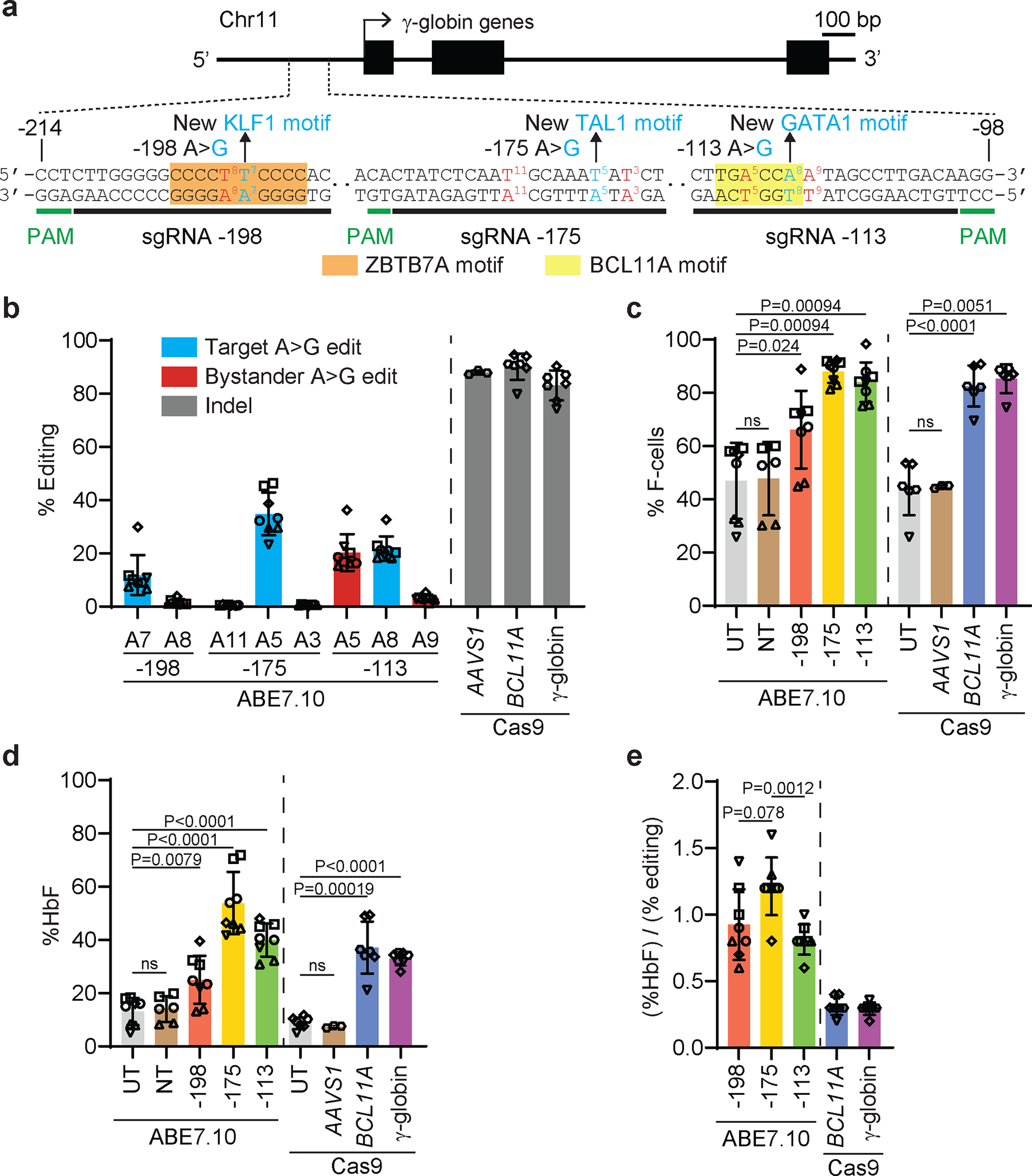
Potent induction of fetal hemoglobin (HbF) with ABE7.10. **a**, The γ-globin gene promoter (*HBG2*; hg-19 chr11:5,276,109–5,276,225, *HBG1*; hg19-chr11:5,271,185–5,271,301) showing variants that cause HPFH by creating new binding motifs for the transcriptional activators KLF1, TAL1, and GATA1. Promoter binding motifs for the transcriptional repressors ZBTB7A and BCL11A are highlighted in orange and yellow, respectively. Single guide RNA (sgRNA) spacer sequences used to install each variant with adenine base editors (ABEs) are represented as black bars, and protospacer-adjacent motifs (PAMs) are represented as green bars. On-target edits and potential bystander edits are in blue and red fonts, respectively, and are numbered from the 5’ end of the protospacer. Healthy donor human CD34^+^ HSPCs were electroporated with ribonucleoprotein (RNP) complex consisting of ABE7.10 complexed with sgRNA −198, ABE7.10-NG complexed with sgRNA −175, or ABE7.10 complexed with sgRNA −113. Alternatively, cells were electroporated with Cas9 complexed with sgRNA targeting the AAVS1 site, the γ-globin promoter BCL11A binding motif, or the *BCL11A* gene erythroid enhancer. Negative controls included untreated (UT) cells or cells transfected with ABE7.10 complexed with non-targeting (NT) sgRNA. Electroporated cells were induced to undergo erythroid differentiation. **b**, Frequencies of on-target and bystander base edits or Cas9 indels determined by next generation sequencing (NGS) on day 3. **c**, %HbF immunostained cells (F-cells) measured by flow cytometry at day 14. **d**, %HbF relative to total hemoglobin in cell lysates, measured by ion-exchange high performance liquid chromatography (HPLC) at day 21. **e**, %HbF normalized to % editing. All bar charts show data as the mean values ± standard deviation (SD). Each symbol shape represents cells from a different donor. Cas9-treated samples (n=7 for UT, *γ-globin* and *BCL11A* for panels b, d and e, n=6 for UT, *γ-globin* and *BCL11A* for panel c, n=3 for *AAVS1* for panels b-e) and ABE-treated samples (n=8 for UT, −198, −175 and −113, n=6 for NT for panels b-e) were measured in separate experiments, indicated by the vertical dashed line. *P* values were calculated using a Wilcoxon rank sum test (panel c) or two-sample *t*-test (panel d and e). ns, not significant.

We electroporated CD34^+^ HSPCs from five healthy donors with ribonucleotide–protein complexes (RNPs) composed of the ABE ABE7.10 protein^13^ and single guide RNAs (sgRNAs) designed to install one of three naturally occurring HPFH mutations that create new binding sites for the erythroid transcriptional activators KLF1 (−198 A>G)^28^, TAL1 (−175 A>G)^29^, or GATA1 (−113 A>G)^30^ (Fig. 1a). Controls included HSPCs that were untreated (UT) or electroporated with RNP containing non-targeting (NT) sgRNA. In separate experiments, we electroporated HSPCs with RNP containing Cas9 with three nuclear localization signals (Cas9–3xNLS, Cas9) and sgRNA targeting a critical GATA1 binding motif in the +58 *BCL11A* erythroid enhancer^31^ (Extended Data Fig. 1a) or the BCL11A binding motif in the γ-globin promoter^8,11^ (Extended Data Fig. 1b). The modified HSPCs were stimulated to undergo erythroid differentiation. On-target base editing frequencies determined after three days by next-generation sequencing (NGS) were 12±7%, 35±8%, and 22±5% at positions −198 (A7), −175 (A5), and −113 (A8), respectively (Fig. 1b). Adjacent to the −113 site (A8), the bystander edit at position A9 should inhibit HbF induction by destroying the GATA motif created by the on-target edit, whereas the A5 bystander edit could augment HbF production by disrupting the BCL11A binding motif. The indel frequencies in cells electroporated with Cas9 RNPs targeting the *BCL11A* erythroid enhancer or the BCL11A binding site in the γ-globin promoter were 90±5% and 83±6%, respectively, with all high-frequency indels predicted to disrupt the targeted regulatory motifs (Fig. 1b, Extended Data Fig. 1c,d). The mean indel rates were <1% after base editing (Extended Data Fig. 1e).

**Extended Data Fig. 2 |:**
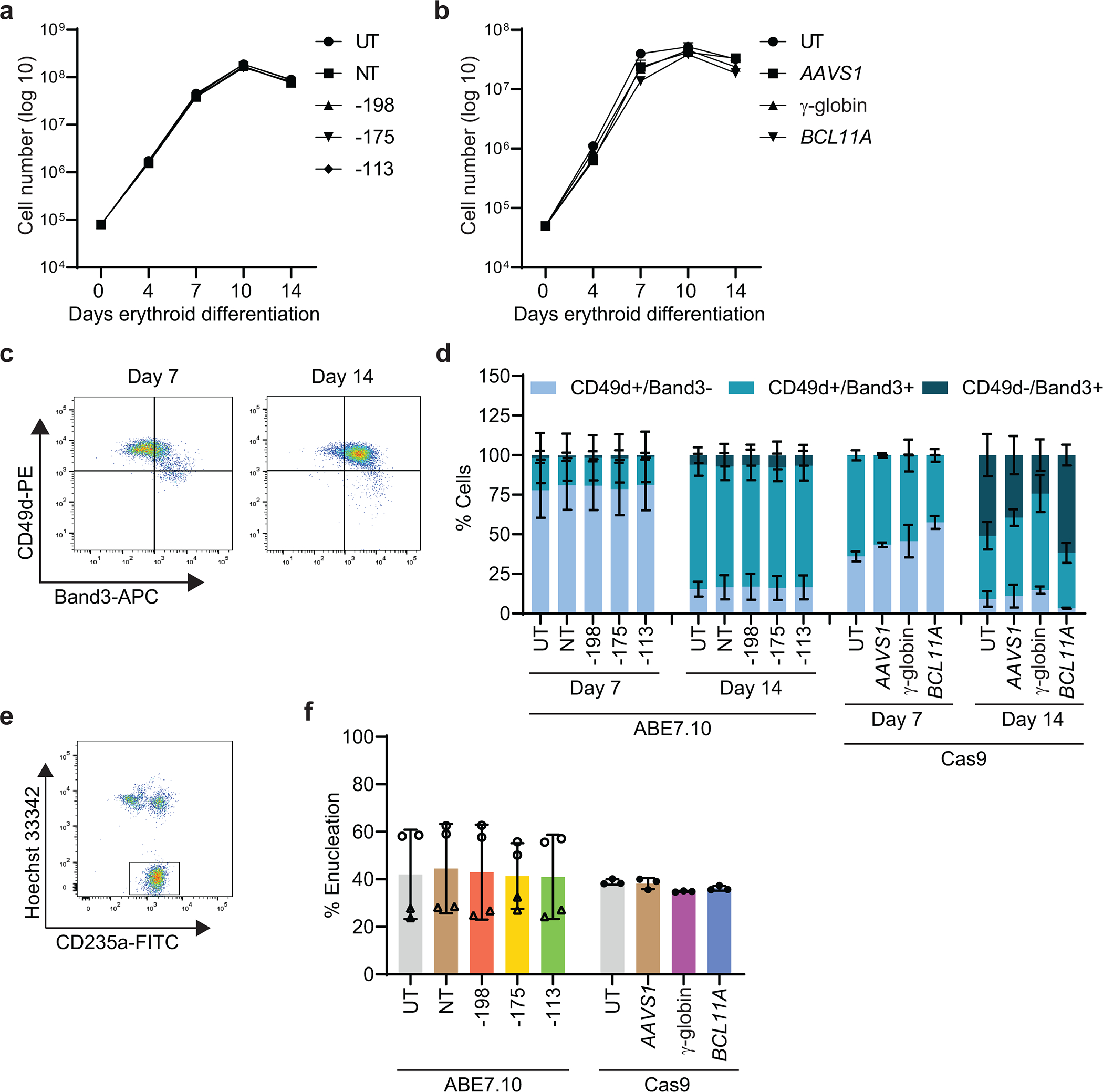
Erythroid differentiation of CD34^+^ HSPCs after editing with ABE7.10 complexed with sgRNA −175 or Cas9 nuclease complexed with sgRNA targeting the BCL11A binding site in the γ-globin promoter, the +58 *BCL11A* erythroid enhancer or the control locus *AAVS1*. Cells were edited by electroporation with RNPs, incubated in CD34^+^ expansion medium for two days, then transferred to erythroid differentiation medium. **a,** Cell expansion after base editing (n=2 from two donors). **b,** Cell expansion after Cas9 editing (n=3 from one donor). **c,** Representative flow cytometry scatter plot showing maturation markers in CD235a^+^ erythroblasts after 7 and 14 days of in vitro erythroid differentiation. **d,** Summary of multiple experiments to assess cell maturation using gating strategy depicted in panel c. n=2 replicates each from two donors for ABE7.10 and n=3 from one donor for Cas9. **e,** Representative flow cytometry scatter plot showing enucleated reticulocytes distinguished by loss of staining with the DNA-binding dye Hoechst 33342 (gated). **f,** Percentage of enucleated CD235^+^ erythroid cells (reticulocytes) at differentiation day 21 (n=4 from two donors for ABE7.10 and n=3 from one donor for Cas9). Graphs show mean ± SD. UT, untreated. NT, non-targeting gRNA.

After in vitro erythroid differentiation, the fraction of HbF immunostaining cells (F-cells) detected by flow cytometry and the %HbF determined by ion-exchange high-performance liquid chromatography (IE-HPLC) of cell lysates were significantly increased in all populations of edited cells, compared to control cells (Fig. 1c,d). There were no differences in expansion or erythroid differentiation in base-edited cells versus control cells (Extended Data Fig. 2a–f). The %HbF per %on-target edit was higher after base editing than after editing with Cas9 and was highest with the –175 A>G edit (Fig. 1e, Supplementary Table 1). A previous study showed that this edit resulted in particularly high induction of HbF in the human erythroid cell line HUDEP2^16^.

**Extended Data Fig. 3 |:**
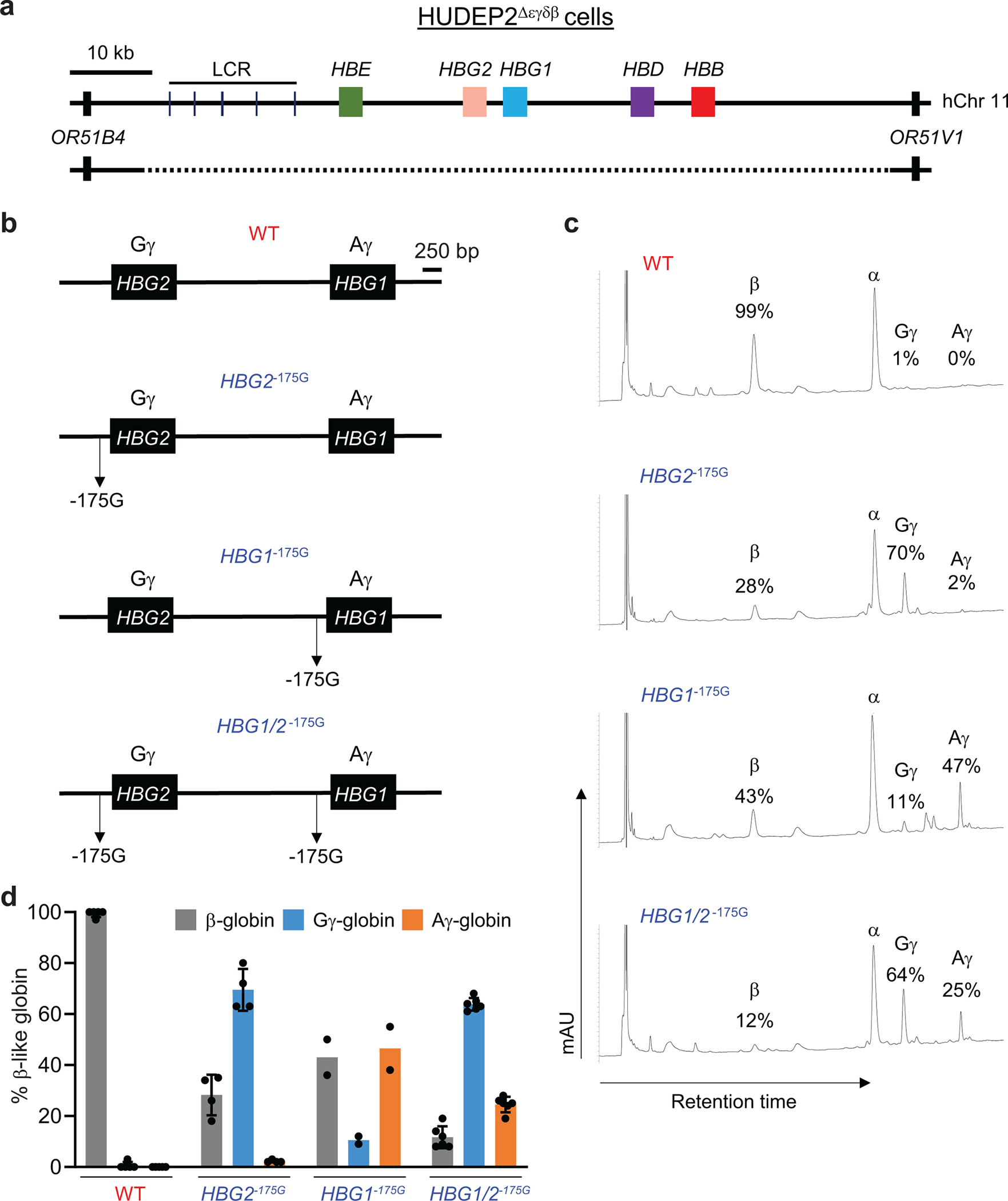
Analysis of γ-globin −175 A>G edited HUDEP2 cells. **a,** HUDEP2^Δεγδβ^ cells harboring a heterozygous 91-kb deletion encompassing the extended β-like globin locus (hg19-chr11:5,219,382–5,324,395) were edited with ABE7.10 protein complexed with sgRNA −175. **b,** Editing outcomes in γ-globin promoters of *HBG2* and *HBG1 (*hg19-chr11:5,269,501–5,276,395) and nomenclature for clones. **c,** Representative reverse-phase high performance liquid chromatography (RP-HPLC) chromatograms of lysates from HUDEP2^Δεγδβ^ clones with the indicated genotypes after 10 days of culture in erythroid differentiation medium. Gγ and Aγ refer to the protein products of *HBG2* and *HBG1*, respectively. **d,** Percentage of β-like globins in HUDEP2^Δεγδβ^ clones with the indicated genotypes. WT, n=5; *HBG2*^−*175G*^, n=4; *HBG1*^−*175G*^, n=2*; HBG1/2*^−*175G*^, n=6. Bar graphs show mean ± SD with each dot representing a separate clone. LCR, locus control region. WT, wild type.

We investigated further the effects of −175 A>G in HUDEP2 cells, which express mainly HbA but can be induced to express HbF by genome editing^11,32–34^. The HUDEP2^Δεγδβ^ line harbors a heterozygous 91-kb deletion of the β-like globin gene cluster (Extended Data Fig. 3a)^34^. We used ABE 7.10 to generate HUDEP2^Δεγδβ^ clones with −175 A>G edits in the promoters of one or both remaining γ-globin genes (Extended Data Fig. 3b). The γ-globin protein products of *HBG*1 and *HBG2*, termed Aγ and Gγ, respectively, differ by one amino acid and can be distinguished by reverse-phase (RP) HPLC (Extended Data Fig. 3c). HUDEP2^Δεγδβ^ clones with wild type γ-globin alleles expressed β-globin exclusively. In contrast, *HBG2*^−175G^ clones expressed 70±8% Gγ and 2±1% Aγ whereas *HBG1*^−175G^ clones expressed 47±12% Aγ and 11±2% Gγ Clones edited at both genes (*HBG1*/*2*^−175G^) expressed 64±2% Gγ and 25±3% Aγ (Extended Data Fig. 3d). The preferential expression of Gγ over Aγ in cells edited at both γ-globin genes is similar to what occurs in normal newborns^35^. These results confirm that the −175 A>G edit potently induces γ-globin expression.

### Reduced DNA damage response and 4.9-kb deletion with base editing.

**Extended Data Fig. 4 |:**
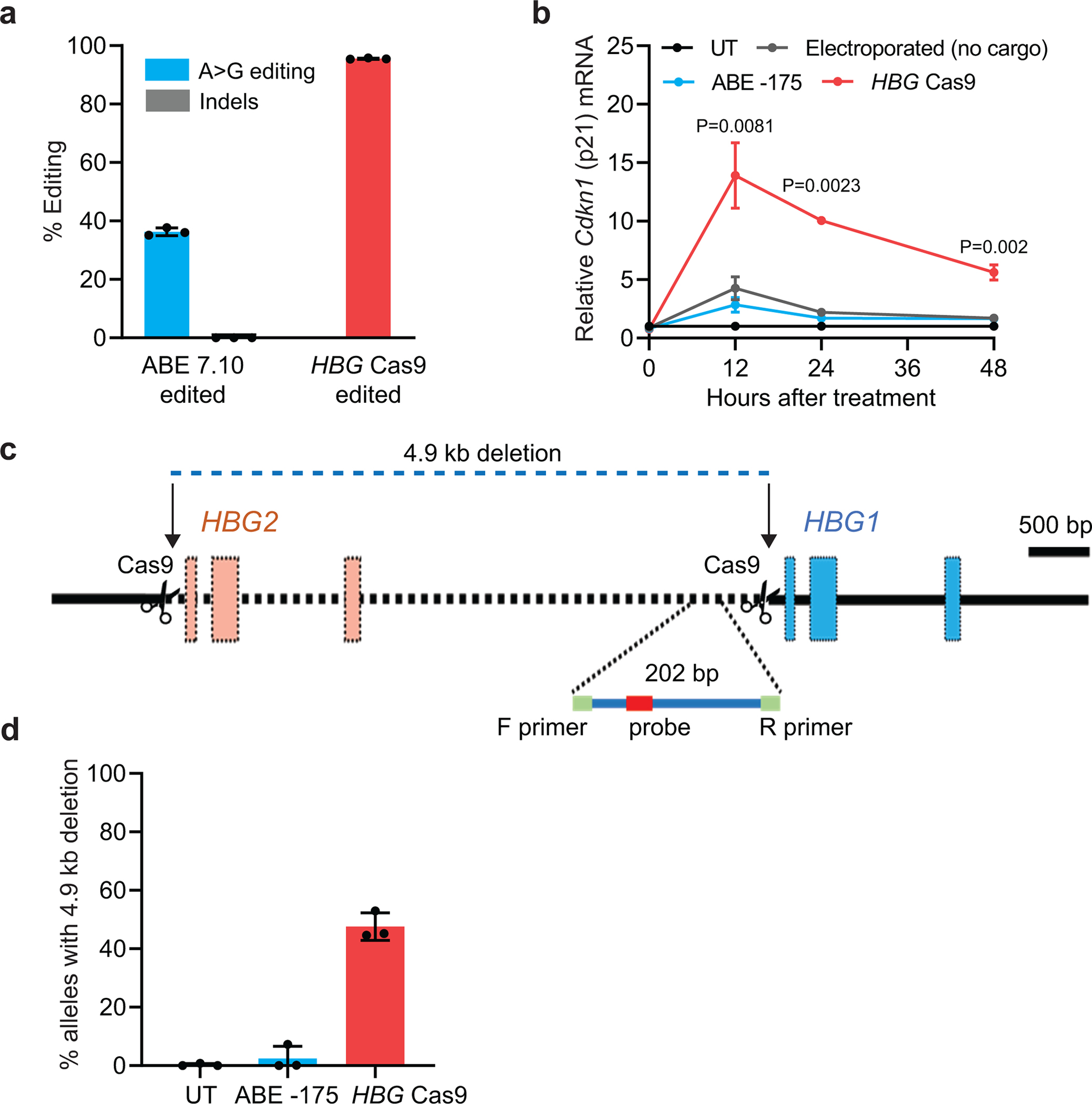
DNA damage response and 4.9-kb deletion analysis of ABE7.10-edited or Cas9 nuclease–edited HSPCs. Healthy donor CD34^+^ cells were edited by electroporation with Cas9 nuclease protein complexed with sgRNA targeting the γ-globin promoter BCL11A binding motif or ABE7.10 protein complexed with sgRNA −175. **a,** Frequencies of on-target ABE7.10 edits or Cas9 nuclease indels measured by NGS. **b,** Relative expression of *CDKN1* (*p21*) mRNA vs. hours after electroporation, measured by droplet digital PCR (ddPCR) and normalized to *RPP30* mRNA. **c,** Quantitative PCR detection method used to assess the frequency of the 4.9-kb *HBG2–HBG1* intergenic deletion resulting from simultaneous on-target indels. **d,** Percentage of the 4.9-kb deletion after editing by ABE7.10 or Cas9 nuclease. Graphs show mean ± SD (n=3 independent replicates from one CD34^+^ cell donor). *P* values were determined using a two-sample *t*-test to assess differences between the Cas9 nuclease-treated samples and the electroporated control. UT, untreated.

**Extended Data Fig. 5 |:**
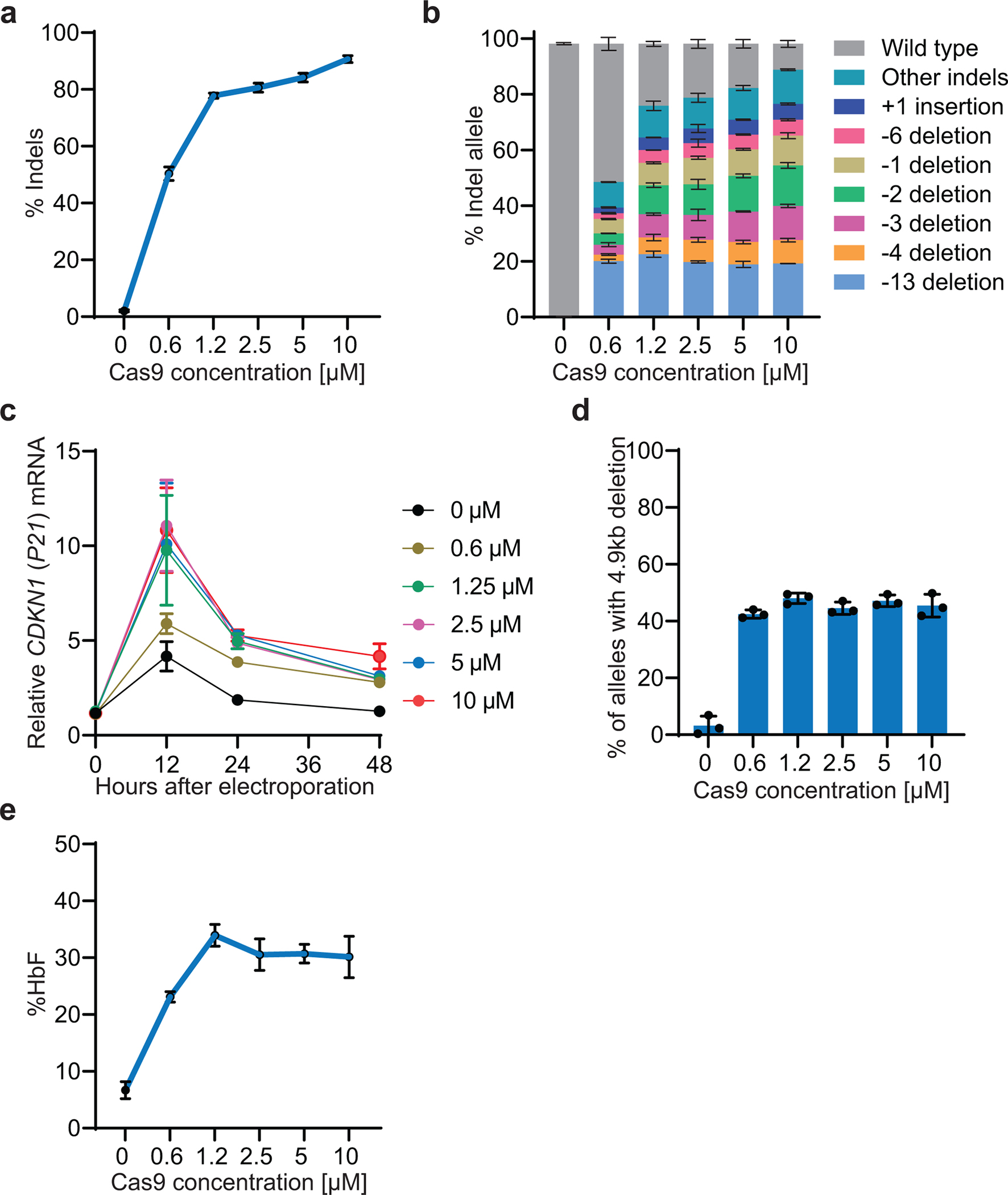
Dose-titration of Cas9 nuclease RNP targeting the BCL11A binding site in the γ-globin promoters. Healthy donor CD34^+^ HSPCs were electroporated with the indicated doses of Cas9 complexed to the sgRNA shown in Extended Data Fig. 1b, followed by induced erythroid differentiation. **a,** Indel frequencies at day 3. **b,** Distributions of specific indels at day 3. **c,** Relative expression of *CDKN1* (*P21*) mRNA vs. hours after electroporation **d,** Percentage of alleles with the 4.9-kb deletion at day 6. **e,** Percentage of HbF on day 21 of erythroid differentiation. Graphs show mean ± SD (n=3 independent replicates from one CD34^+^ cell donor).

Cas9 disruption of the BCL11A binding site in the γ-globin promoter of healthy-donor CD34^+^ HSPCs resulted in >90% indels with transient induction of the TP53-mediated DNA damage response, as indicated by increased expression of the *CDKN1* (p21) gene^36,37^ (Extended Data Fig. 4a,b), whereas ABE7.10 installation of the −175 A>G variant resulted in *CDKN1* mRNA levels similar to those in control cells. Simultaneous nuclease cleavage of both tandem *HBG1* and *HBG2* genes by Cas9 can result in deletion of the intervening 4.9-kb sequence, generating a single functional *HBG2-HBG1* fusion gene (Extended Data Fig. 4c)^8^. This occurred in 48±5% of alleles after Cas9 disruption of the BCL11A binding site in the γ-globin promoter but was not detected after ABE7.10 editing (Extended Data Fig. 4d). We obtained similar results with Cas9 RNP concentrations between 1.2 and 10μM (Extended Data Fig. 5a–e).

### The γ-globin −175 A>G variant generates a functional GATA-TAL1 binding motif.

**Extended Data Fig. 6 |:**
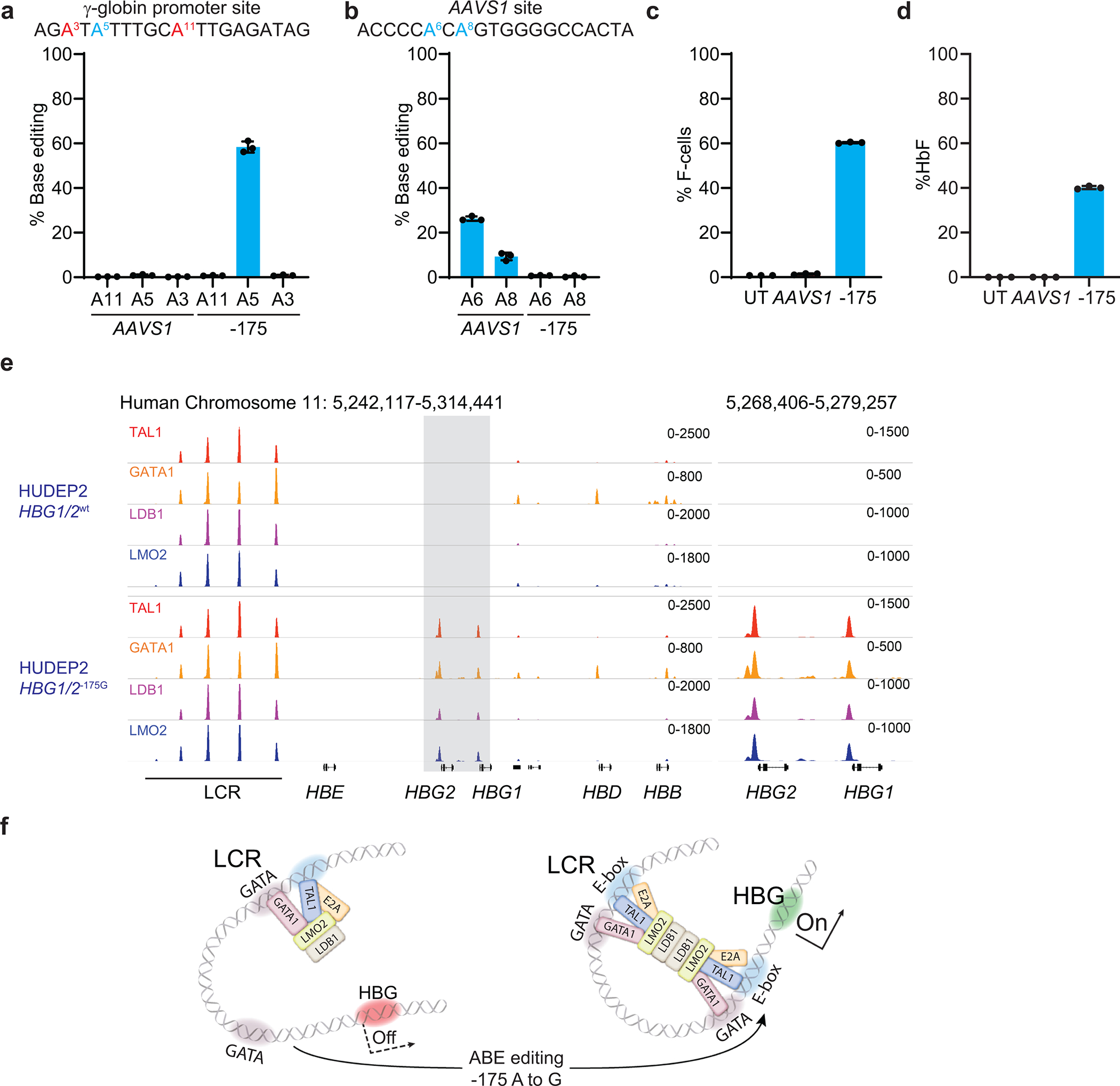
Effect of the −175 A>G edit on HbF and transcription factor binding in WT HUDEP2 cells (β-globin-like globin loci intact). HUDEP2 cells were electroporated with ABE7.10 protein complexed with sgRNA −175. Negative controls included untreated cells (UT) that received no electroporation and cells electroporated with ABE7.10 protein complexed with AAVS1 sgRNA. **a,** Frequencies of on-target (A5) and bystander (A3, A11) γ-globin edits 3 days after electroporation. **b,** Frequencies of AAVS1 edits at 3 days after electroporation. **c,** % F-cells in the bulk edited population on day 4 after electroporation. **d,** %HbF in the bulk-edited population after 10 days of erythroid maturation. Graphs show mean ± SD (n=3). **e,** CUT&RUN analysis to assess chromatin occupancy of TAL1, GATA1, LDB1, and LMO2 in a wild type (WT) HUDEP2 clone or a clone with −175 A>G base edits at all four γ-globin promoters. **f,** Model for HbF induction by −175 A>G. The −175 A>G variant creates a new TAL1 binding motif near a GATA motif that binds GATA1. Binding of TAL1 stimulates recruitment of the indicated proteins. Homodimerization of LDB1 within the γ-globin promoter protein complex and a similar complex at the locus control region (LCR) mediates DNA looping and transcriptional activation. WT, wild type; UT, untreated.

**Fig. 2 |:**
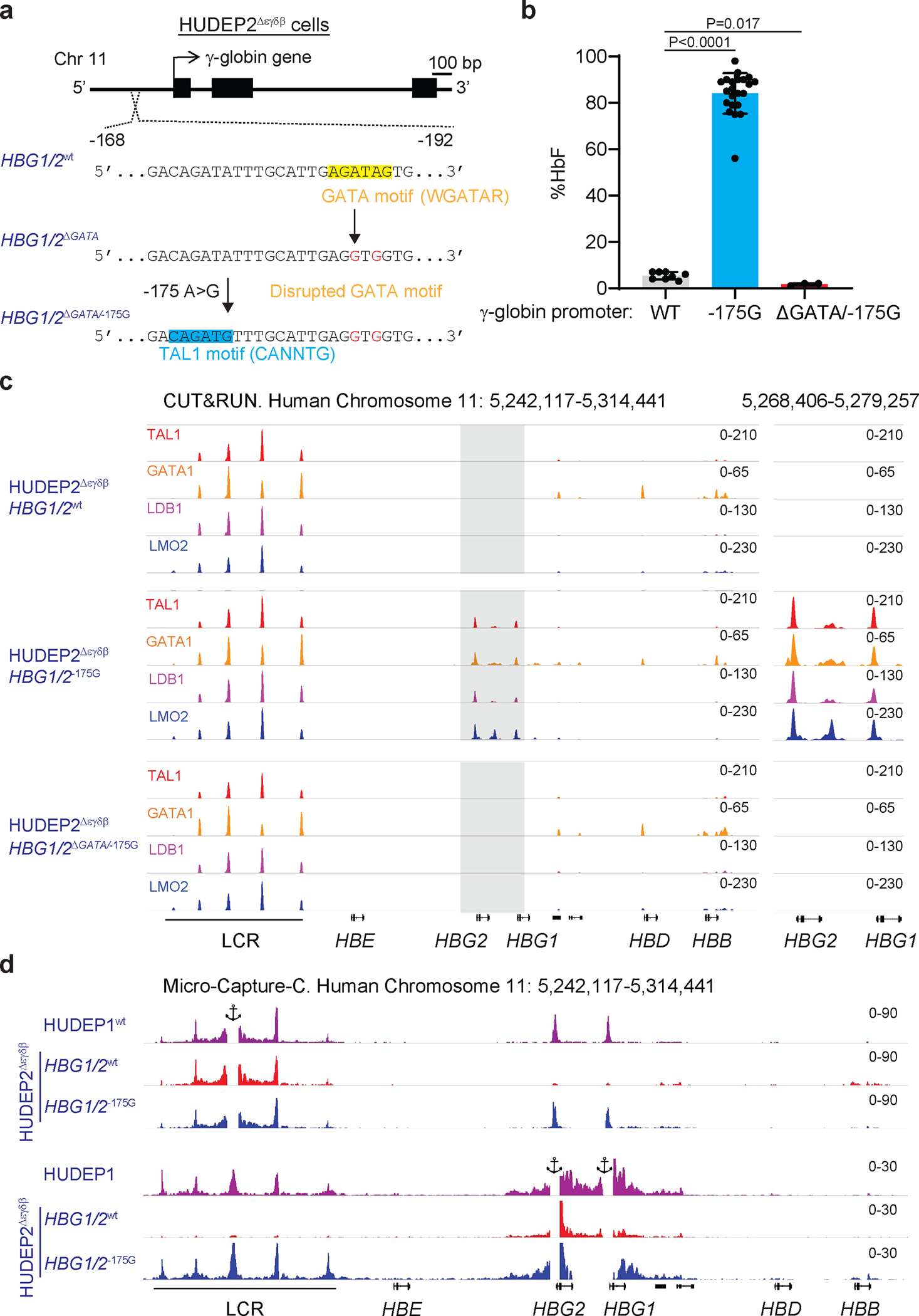
γ-globin −175 A>G generates a TAL1-GATA1 binding motif that stimulates promoter–enhancer interactions. **a,** Mutational analysis of the composite motif (*HBG2;* hg19-chr11:5,276,179–5,276,203, *HBG1*; hg19-chr11:5,271,255–5,271,279) in HUDEP2^Δεγδβ^ cells, which are heterozygous for a 91-kb deletion of the β-like globin gene cluster (Extended Data Fig. 3a). The GATA motif, highlighted in yellow, was disrupted by ABE7.10 to generate ΔGATA, followed by ABE7.10-NG installation of −175 A>G, which creates a TAL1 motif, shown in blue. Individual clones were isolated and analyzed after 10 days of induced erythroid maturation. **b**, %HbF in HUDEP2^Δεγδβ^ cells with the indicated γ-globin promoter genotypes. Each dot represents an individual mutant clone. The bar chart shows the mean ± SD with *P* values calculated using a Wilcoxon rank sum test. WT, n=8; −175G, n=22; ΔGATA/−175G, n=3. **c**, CUT&RUN analysis to assess chromatin occupancy of TAL1, GATA1, LDB1, and LMO2. The gray highlighted region is magnified at right. **d**, Micro-Capture-C analysis to identify long-range chromatin interactions in HUDEP1 cells, which express mainly HbF thus serving as a positive control, and in HUDEP2^Δεγδβ^ clones with the indicated genotypes at both γ-globin promoters (*HBG1* and *HBG2*). Panels show the results experiments with anchors at the LCR HS3 region or the γ-globin promoter. WT, wild type.

The −175 A>G edit creates a binding motif for the transcription factor TAL1 (CANNTG) located 9 bp downstream of a functionally important GATA1 binding motif (WGATAR)^34^. Binding of TAL1 and GATA1 to this compound motif recruits the LIM domain only 2 (LMO2) protein and LIM domain binding protein 1 (LDB1), which mediate long-range interactions between β-like globin genes and the LCR^29,39^. We used ABE7.10 to disrupt the upstream GATA motif (A>G, positions −187 and −189) in HUDEP2^Δεγδβ^ cells, which contain a heterozygous disruption of the extended β-like globin locus (Fig. 2a). Clones harboring −175 A>G at both γ-globin genes (*HBG1/2*^−175G^) expressed 84±9% HbF versus 5±2% HbF in unedited control clones (*HBG1/2*^WT^) (Fig. 2b). The −175G variant failed to induce HbF when the adjacent GATA motif was disrupted (*HBG1/2*^ΔGATA/−175G^) (Fig. 2b). CUT&RUN analysis revealed GATA1, TAL1, LMO2, and LDB1 occupancy at the LCR in HUDEP2^Δεγδβ^ cells for all γ-globin genotypes examined (Fig. 2c). In contrast, GATA1/TAL1/LMO2/LDB1 occupancy was detected at the γ-globin promoters in *HBG1/2*^−175G^ clones, but not in *HBG1/2*^WT^ or *HBG1/2*^ΔGATA/−175G^ clones. We obtained similar CUT&RUN results in HUDEP2 cells with intact biallelic β-like globin loci when −175 A>G was present in all four γ-globin promoters (Extended Data Fig. 6a–e). Micro-Capture-C analysis showed that the −175 A>G variant stimulated long-range interaction between the γ-globin promoters and the LCR (Fig. 2d). Thus, the −175 A>G variant creates a bipartite GATA–TAL1 motif, causing the assembly of a GATA1/TAL1/LMO2/LDB1 complex that activates γ-globin gene transcription by enhancing its physical association with the LCR^29^ (Extended Data Fig. 6f).

### Clonal analysis of editing outcomes

**Fig. 3 |:**
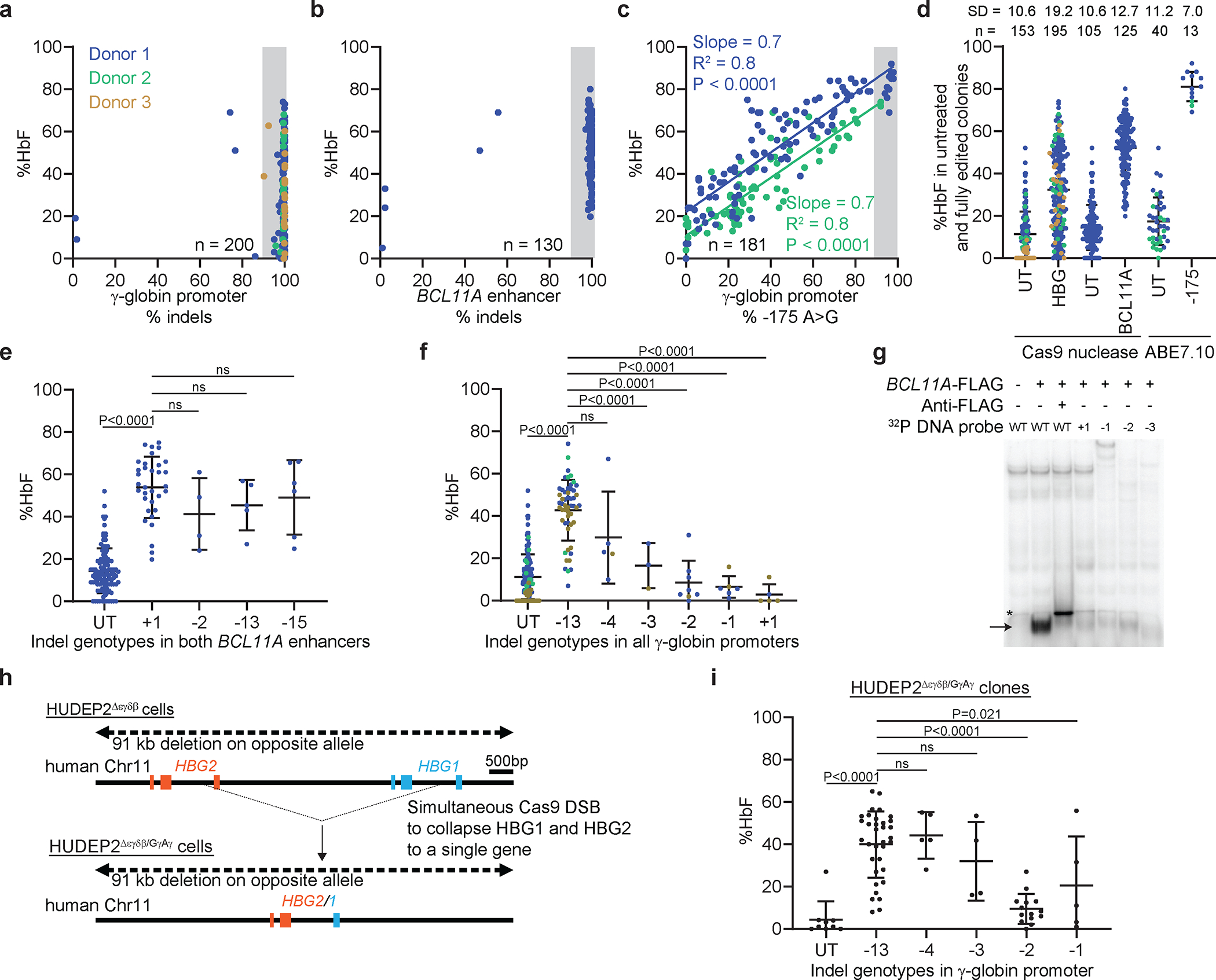
Base editing induces HbF more potently and with less clonal variability when compared with Cas9. In panels a-f and i, healthy-donor CD34^+^ HSPCs were electroporated with RNPs consisting of Cas9 complexed with sgRNA targeting the *BCL11A* binding motif in the γ-globin promoter or the *BCL11A* erythroid enhancer, or with ABE7.10-NG complexed with sgRNA −175. After 3 days, the cells were transferred into methylcellulose medium. Erythroid colonies were isolated and analyzed on day 14. **a–c**, %HbF vs. % on-target edit. Gray highlighting indicates colonies with ≥87.5% edits (“fully edited“). **d**, %HbF of fully edited colonies shown in a–c, side by side with UT controls assessed simultaneously with each editing strategy. The SD of the %HbF between colonies for each editing strategy is shown above. **e**, %HbF in colonies homozygous for the same BCL11A erythroid enhancer indel (UT, n=105; +1, n=33; −2, n=4; −13, n=5; −15, n=6). f, %HbF in colonies with the same indel in all γ-globin promoters (UT, n=153; −13, n=53; −4, n=5; −3, n=3; −2, n=8; −1, n=6; +1, n=5). **g**, Gel-shift assay results. COS cell extract expressing a FLAG tag fused to zinc fingers 4–6 of BCL11A was incubated with radiolabeled WT or mutant probes representing the γ-globin BCL11A binding motif, separated on a polyacrylamide gel and analyzed by autoradiography. Arrow shows the position of BCL11A bound to WT probe; * denotes antibody-supershifted complex. **h**, HUDEP2^Δεγδβ^ cells were edited to generate HUDEP2^Δεγδβ/Gγ–Aγ^ cells, which contain a single functional *HBG2–HBG1* (Gγ–Aγ) fusion gene (Gγ hg19-chr11:5,269,435–5,269,886, Aγ hg19-chr11:5,275,238–5,276,077). i, %HbF in HUDEP2^Δεγδβ/Gγ–Aγ^ clones harboring specific indels in the γ-globin promoter BCL11A binding motif (UT, n=9; −13, n=34; −4, n=5; −3, n=4; −2, n=14; −1, n=5). For panels a-f, each dot represents an individual colony, and each color represents a different donor. For panel c, the slope (coefficient estimate), the coefficient of multiple determination (R^2^), and *P* values were calculated based on the linear regression model. Panels d-f and i shows the mean ± SD with *P* values (e, f, and i) calculated based on the linear regression model adjusting for batch effects. UT, untreated; ns, not significant.

We electroporated CD34^+^ HSPCs with RNPs consisting of ABE7.10 targeting γ-globin −175 or Cas9 targeting either the *BCL11A* erythroid enhancer, or Cas9 targeting the γ-globin promoter BCL11A binding site (Fig. 1a, Extended Data Fig. 1a, b), and generated erythroid colonies. The Cas9 indel frequencies were 100% in most colonies, although the HbF levels varied greatly (Fig. 3a,b). In contrast, the −175 A>G base editing efficiency was lower and the HbF levels correlated with the editing frequency (Fig. 3c, Supplementary Table 2). These findings occurred with HSPCs from two different donors, although the extent of HbF induction differed slightly between donors^8,40^. In colonies with ≥87.5% on-target editing (shaded regions in Fig. 3a–c), the −175 A>G edit resulted in a higher %HbF with less variability when compared with the %HbF resulting from Cas9 editing (Fig. 3d, Supplementary Table 3).

**Extended Data Fig. 7 |:**
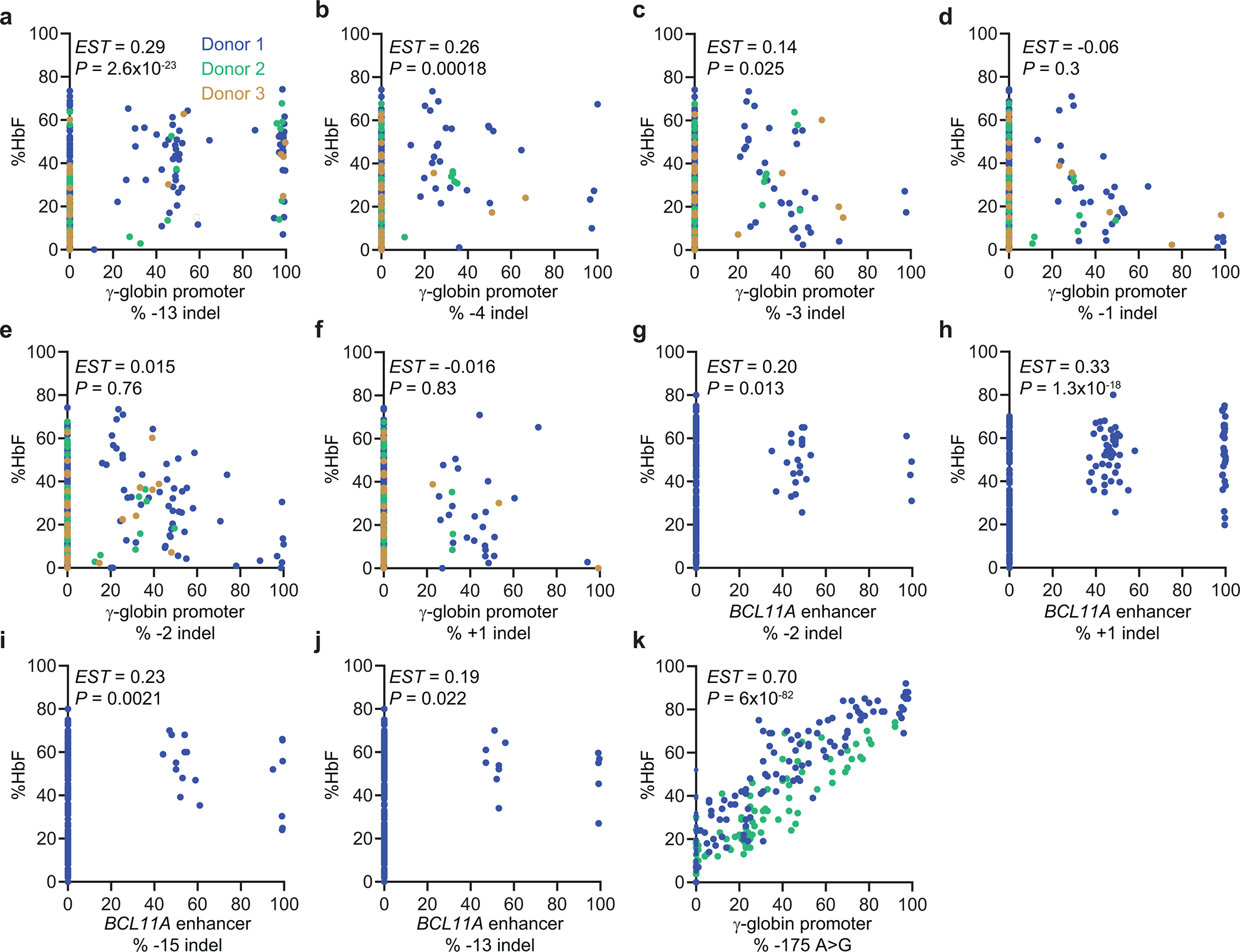
Induction of HbF according to the frequency of common indels alleles in erythroid colonies generated from Cas9 nuclease-edited or base edited CD34^+^ cells. **a–f,** Percentage of HbF according to the frequency of the specified indel in the γ-globin promoter BCL11A motif. Most colonies analyzed have indels at all γ-globin promoters (see main Fig. 3a, b). Colonies have 0% of a given indel allele if the indicated indel was not detected by sequencing that colony (n=353). **g–j,** Percentage of HbF according to the frequency of the specified indel in the +58 *BCL11A* erythroid gene enhancer (n=228). **k,** Percentage of HbF according to the frequency of the ABE7.10-generated −175 A>G (n=221). EST, coefficient estimate, corresponding to the slope of the linear regression line, adjusting for batch effects; P, statistical significance calculated by a linear regression model adjusting for batch effects. Each dot represents a separate clone and each color represents a different CD34^+^ cell donor.

We next investigated the HbF levels associated with the most common indels in the BCL11A erythroid enhancer or in the γ-globin promoter BCL11A motif (Extended Data Fig. 1a,b). All high-frequency indels targeting the *BCL11A* erythroid enhancer disrupted the functionally important GATA1 binding motif^31^ and induced HbF to similarly (Fig. 3e, Extended Data Figs. 1a,c, 7g–j, Supplementary Table 4), although the %HbF varied considerably between colonies that were homozygous for the most common indel (+1) (Fig. 3e). In contrast, HbF induction varied according to different Cas9 indels in the BCL11A binding motif of the γ-globin promoter. Specifically, the −13-bp and −4-bp deletions were associated with a higher %HbF than were the −1-bp, −2-bp, or −3-bp deletions, or +1-bp insertion (Fig. 3f), despite similar interference with BCL11A binding (Fig. 3g). Similarly, in multivariate linear regression analysis of colonies with heterogeneous on-target indels, HbF induction correlated with the number of −13-bp, −4-bp, and −3-bp indel alleles but not with the −1-bp, −2-bp, or +1-bp indel alleles (Extended Data Fig. 7a–f, Supplementary Tables 4,5). To investigate further the relations between specific indels and HbF induction, we used Cas9 to mutate the γ-globin promoter in HUDEP2^Δεγδβ/GγAγ^ cells, which harbor a single γ-globin gene (Fig. 3h). The HUDEP2^Δεγδβ/GγAγ^ clones with the −13-bp deletion exhibited higher %HbF than clones with the −2-bp deletion (Fig. 3i). The %HbF also tended to be lower with the −1-bp and −3-bp deletions, although relatively few clones were identified, and the differences were not significant.

Overall, clonal analysis showed that Cas9 indels at the γ-globin promoter BCL11A binding site or at the *BCL11A* erythroid enhancer result in HbF induction that varies between clones, possibly due to indel-specific activities or to stochastic differences between clones with the same indel. The −175 A>G base edit resulted in more potent and consistent induction of HbF.

### Improved HSPC base editing with ABE8e

**Extended Data Fig. 8 |:**
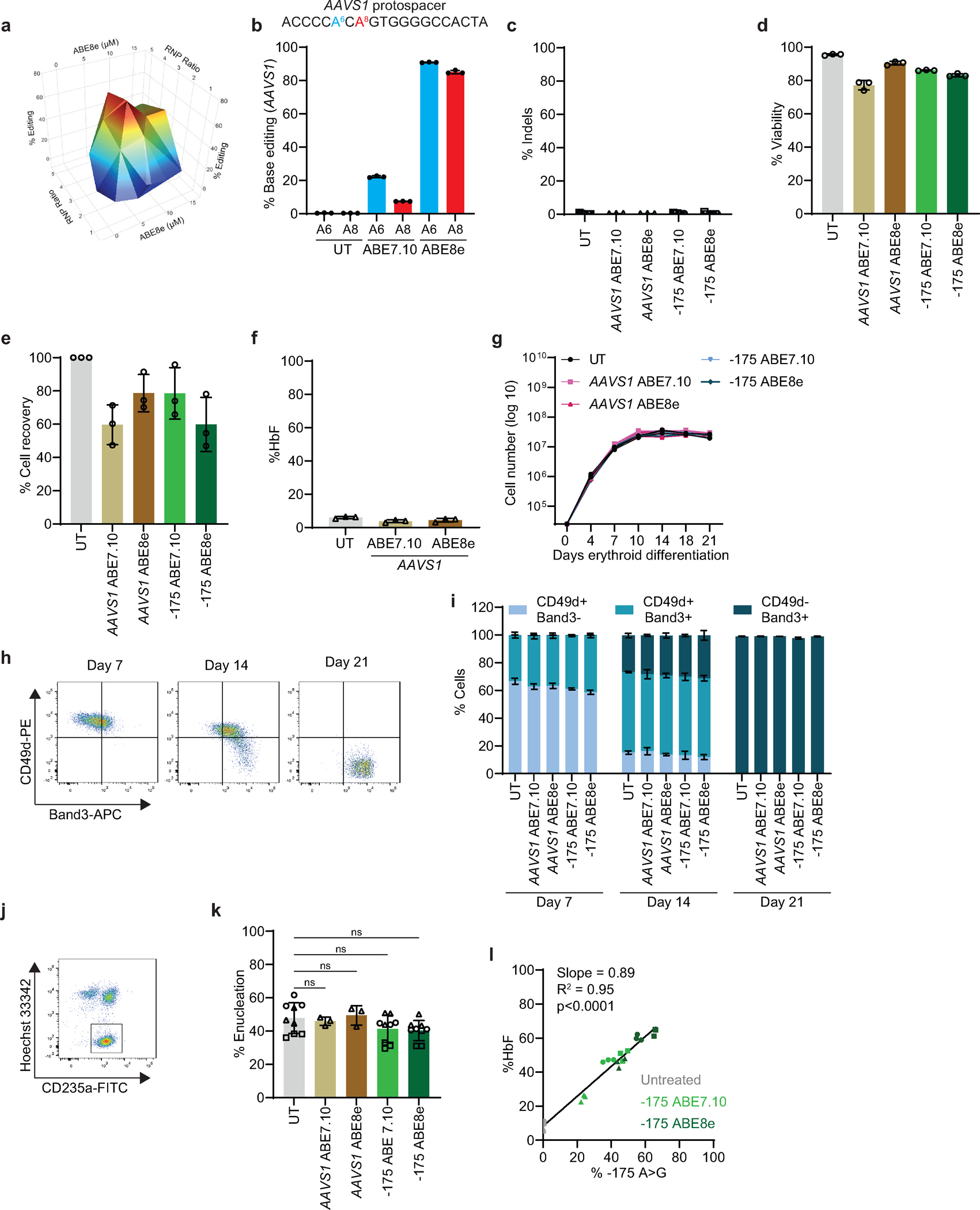
Comparison of ABE7.10 and ABE8e editing at γ-globin −175 A>G in healthy donor CD34^+^ HSPCs. Controls included UT and *AAVS1* sgRNA. Cells were edited by electroporation with RNPs, incubated in expansion medium for two days, then transferred to erythroid differentiation medium. **a,** Results of a Design of Experiment (DoE) study to optimize ABE8e concentration and sgRNA molar ratio (see Methods). Red color indicates most efficient editing. An ABE concentration of 8 μM with a 3.5-fold excess of sgRNA was determined to be optimal and used in subsequent experiments. **b**, AAVS1 editing frequencies six days after electroporation (n=3). **c,** Indel frequencies six days after electroporation (n=9 for UT, ABE7.10 and ABE8e −175; n=3 for AAVS1, ABE7.10 and ABE8e). **d**, Cell viability and **e,** cell recovery two days after electroporation (n=3). **f,** %HbF in control edited cells (n=3). **g**, Cell number versus days erythroid differentiation (n=3). **h,** Representative flow cytometry scatter plots of maturation markers in CD235a^+^ erythroblasts after 7 and 14 days of in vitro erythroid differentiation. **i,** Summary of multiple experiments to assess cell maturation using gating strategy depicted in panel h. n=3 replicates from one CD34+ cell donor. **j,** Representative flow cytometry scatter plot showing enucleated reticulocytes distinguished by loss of staining with the DNA-binding dye Hoechst 33342 (gated). **k,** Percentage of enucleated CD235^+^ erythroid cells (reticulocytes) at differentiation day 21 (n=9 for UT, ABE7.10 and ABE8e −175; n=3 for AAVS1, ABE7.10 and ABE8e). **l,** Percentage of HbF versus editing frequencies. A linear regression model was used to correlate %HbF with %−175 A>G in panel l (n=9). Each symbol represents a different donor. Graphs show mean ± SD. The slope (coefficient estimate), coefficient of multiple determination (R^2^), and *P* values were calculated based on two sample *t*-test (panel k) and a linear regression model (panel l). UT, untreated; ns, not significant.

**Fig. 4 |:**
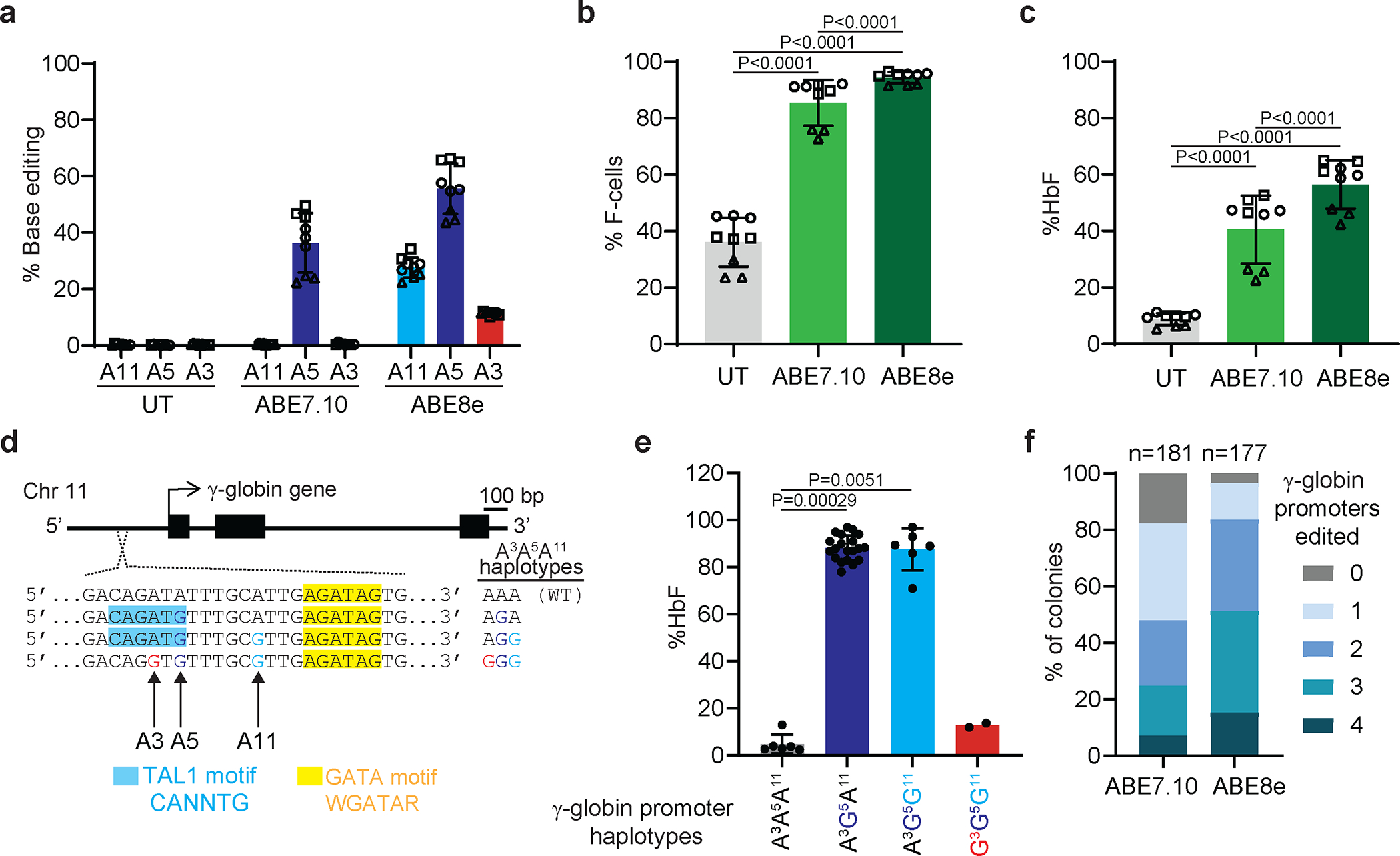
Improved −175 A>G editing frequency and HbF induction with ABE8e. For experiments shown in panels a-c and f, healthy donor CD34^+^ cells were electroporated with RNPs consisting of ABE7.10 or ABE8e protein complexed with sgRNA −175 followed by in vitro erythroid differentiation. **a**, Frequencies of on-target (A5) and bystander (A3, A11) edits 6 days after electroporation. **b**, % F-cells on day 14. **c**, %HbF on day 21. (n=9, from 3 donors). Each symbol represents a separate experiment with different symbols designating unique donors. **d**, Common haplotypes generated by on-target and bystander editing. e, %HbF in ABE8e-edited HUDEP2^Δεγδβ^ cell clones with the indicated haplotypes at both γ-globin promoters. A^3^A^5^A^11^, n=6; A^3^G^5^A^11^, n=20; A^3^G^5^G^11^, n=6; G^3^G^5^G^11^, n=2. **f**, Percentages of CD34+ HSPC-derived erythroid colonies with zero to four productive on-target γ-globin promoter edits (A5, not A3) after installation of −175 A>G with ABE7.10 or ABE8e. Bar charts show the mean ± SD with *P* values calculated by a linear regression model adjusting for donor effects (panels b and c) and by a Wilcoxon rank sum test (panel e). UT, untreated; ns, not significant.

We compared ABE7.10 to the newer, more active ABE8e^41^. We optimized the ABE8e concentration and its stoichiometry with sgRNA to install the γ-globin −175 A>G edit (Extended Data Fig. 8a). In CD34^+^ HSPCs from three healthy donors, the editing efficiency was 36±11% with ABE7.10 and 56±9% with ABE8e (Fig. 4a). Editing frequencies at the AAVS1 locus were also greater for ABE8e than for ABE7.10 (Extended Data Fig. 8b), with no detectable indels (Extended Data Fig. 8c). Cell viability and recovery were similar after editing with ABE7.10 or ABE8e (Extended Data Fig. 8d,e). After in vitro erythroid differentiation, the %F-cells rose from 36±9% in untreated control cells to 85±8% or 94±2% after editing at γ-globin −175 with ABE7.10 or ABE8e, respectively (Fig. 4b). The %HbF increased from 9±2% in control cells to 41±12% after editing with ABE7.10 or 56±9% after editing with ABE8e (Fig. 4c, Extended Data Fig. 8f). No abnormalities in erythroid cell expansion or maturation were detected after editing with either ABE (Extended Data Fig. 8g–k).

Overall, HbF induction correlated with γ-globin −175 A>G editing by ABE7.10 or ABE8e (Extended Data Fig. 8l). However, ABE8e produced a higher frequency of bystander edits: 11±1% at position A3 and 28±4% at A11 (Fig. 4a). We studied the impact of these bystander edits in HUDEP2^Δεγδβ^ cells (Extended Data Fig. 3a) by generating ABE8e-edited clones in which both γ-globin genes *(HBG1* and *HBG2)* contained the on-target A5 edit ± bystander edits (Fig. 4d, e). The A5 edit alone (haplotype A^3^G^5^A^11^ at *HBG1* and *HBG2*) induced high-level HbF that was not altered by a concomitant A11 edit (A^3^G^5^G^11^). In contrast, both bystander edits together with the on-target edit (G^3^G^5^G^11^) caused a significant reduction in the %HbF. Thus, the A3 edit likely interferes with HbF induction by −175 A>G, consistent with findings that C or A are favored over G or T at the corresponding position in the TAL1 binding motif (CANNTG)^42,43^. In CD34^+^ cell-derived erythroid colonies, the frequencies of one or more productive haplotype (A^3^G^5^A^11^ or A^3^G^5^G^11^, not G^3^G^5^G^11^) were greater after treatment with ABE8e than with ABE7.10 (Fig. 4f, Supplementary Tables 2 and 6). Therefore, the higher editing efficiency of ABE8e outweighs the negative effects of A3 bystander editing for HbF induction.

### Durable editing of HSCs with induction of HbF in erythroid progeny

**Extended Data Fig. 9 |:**
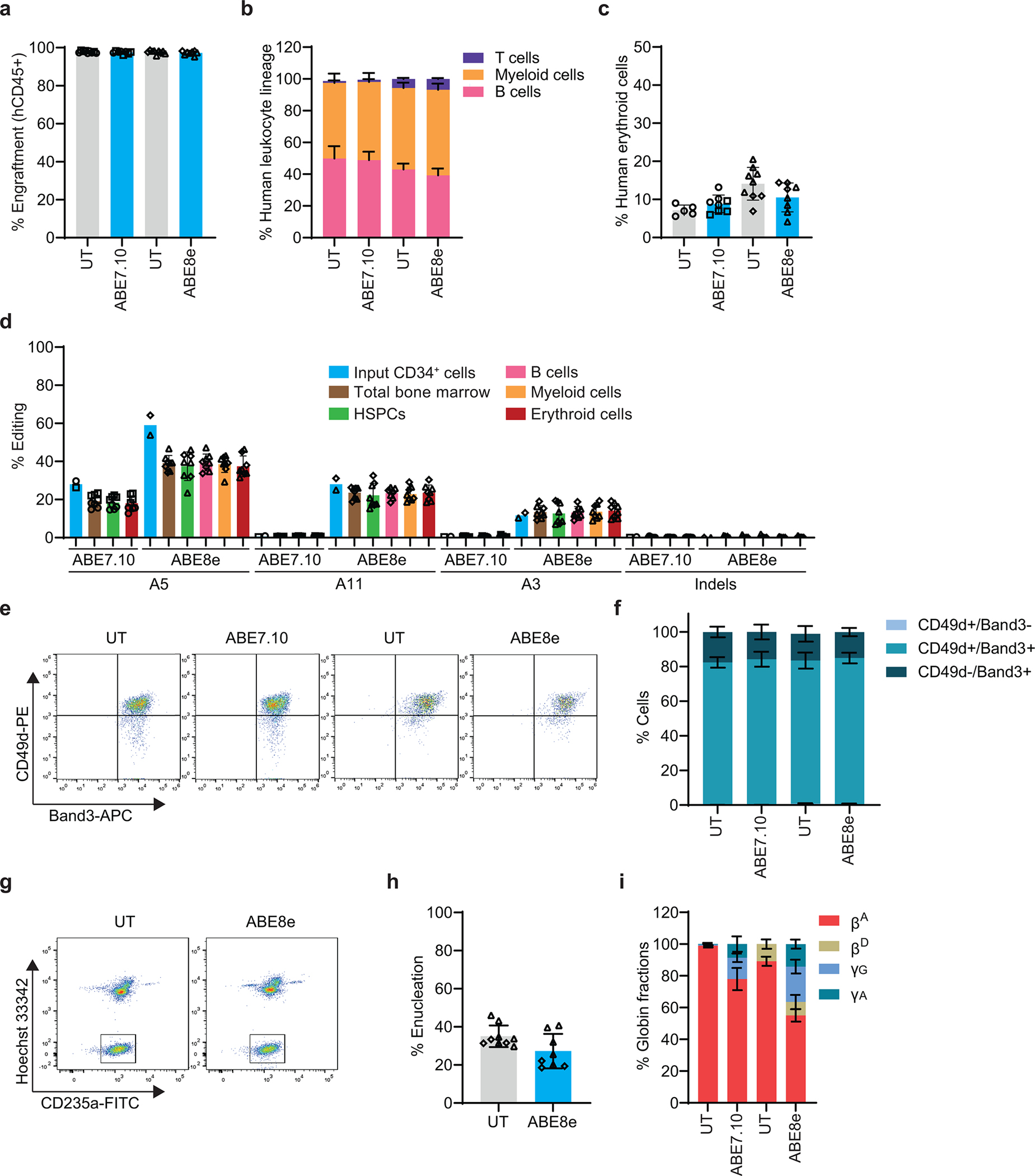
Durable editing in HSCs. **a,** Percentage of human CD45^+^ (hCD45^+^) donor cells in mouse bone marrow 16 weeks after transplantation. **b,** Percentages of B cells, myeloid cells, and T cells within the hCD45^+^ population from mouse bone marrow. **c,** Percentage of hCD235a^+^ erythroid cells in mouse bone marrow. **d,** On-target edits, bystander base edits, and indel frequencies in bone marrow and subpopulations. **e,** Representative flow cytometry scatter plot of maturation markers in CD235a^+^ erythroblasts in bone marrow. **f,** Summary of multiple experiments to assess erythroid cell maturation using gating strategy depicted in panel e. **g,** Representative flow cytometry scatter plot showing enucleated human reticulocytes in mouse bone marrow, distinguished by loss of staining with the DNA-binding dye Hoechst 33342 (gated). **h,** Percentage of enucleated hCD235^+^ erythroid cells (reticulocytes). **i,** The percentage β-like globin proteins in hCD235a^+^ erythroid cells recovered from mouse bone marrow. For the experiments assessing ABE7.10, n=9 mice in the untreated samples and n=8 mice in the ABE7.10 treated samples, corresponding to three to five mice transplanted with cells from each of two healthy donors. For the experiments assessing ABE8e, n=9 mice in the untreated samples and n=8 mice in the ABE8e treated samples, corresponding to three to six mice transplanted with cells from each of two healthy donors. All graphs show mean ± SD. Each symbol shape represents cells from a different CD34^+^ cell donor. UT, untreated. ns, not significant.

**Fig. 5 |:**
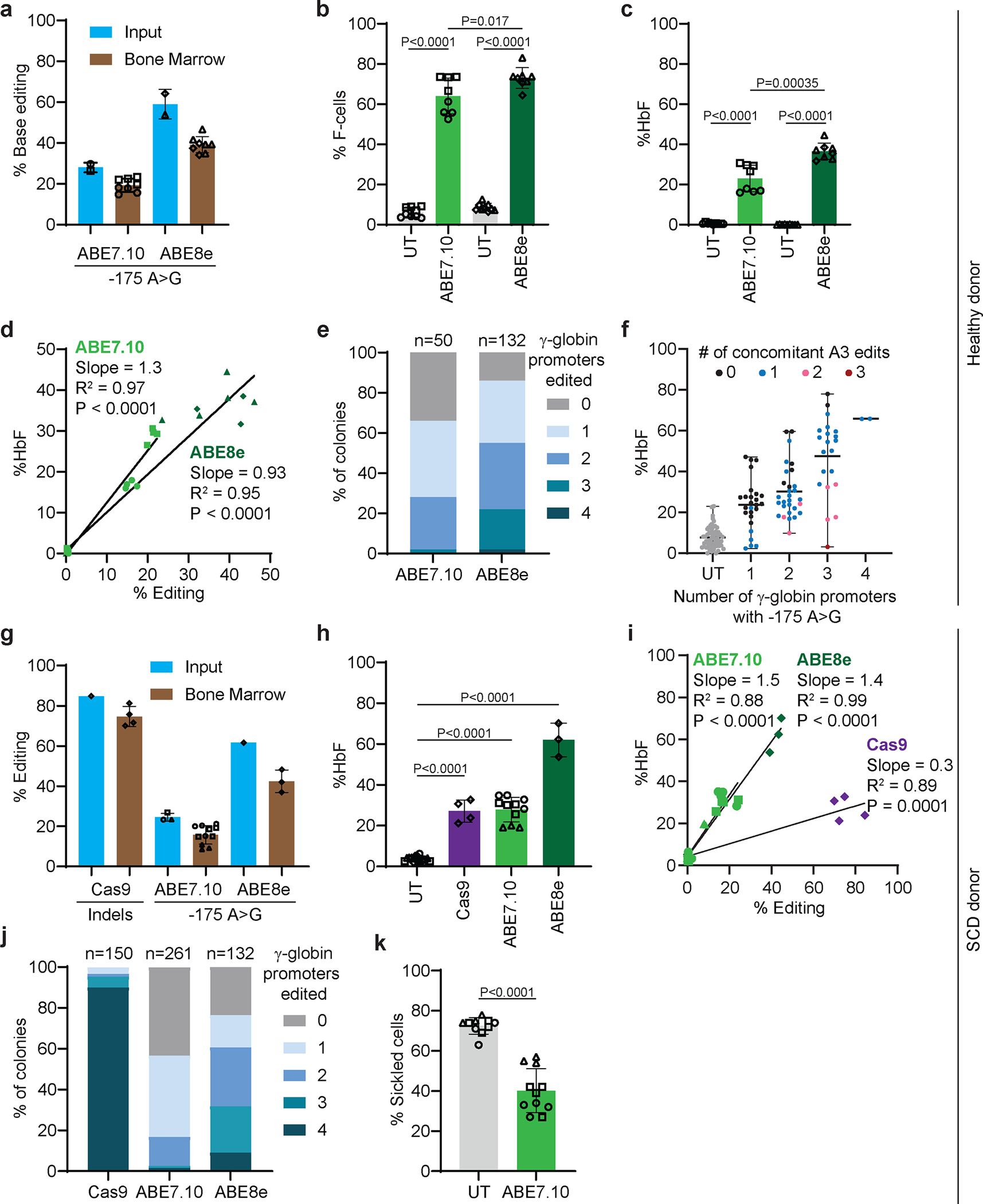
Durable base editing of γ-globin −175 A>G in bone marrow–repopulating HSCs with induction of HbF in erythroid progeny. CD34^+^ HSPCs from healthy (a–f) or SCD (g–k) donors were electroporated with RNP consisting of ABE7.10 or ABE8e with sgRNA −175, or Cas9 with sgRNA targeting the BCL11A binding site of the γ-globin promoter, then transplanted into NBSGW mice. After 16 weeks, mouse bone marrow (BM) was analyzed for human donor–derived cells. **a**, Frequencies of on-target (A5) editing 3 days after electroporation (input, n=2) and in donor-derived cells in BM after transplantation (n=8). **b-d**, Human CD235^+^ (hCD235^+^) erythroblasts in BM analyzed for %F-cells (b), %HbF (**c**), and %HbF vs. on-target editing efficiency (**d**) (UT, n=9; ABE7.10 and ABE8e, n=8). **e**, Percentages of BM–derived human erythroid colonies with zero to four productive on-target edits (A5, not A3). **f**, %HbF according to the number of γ-globin promoters with the −175 A>G edit (A5). Each dot represents an individual colony, with colors indicating the number of A3 bystander edits per colony (UT, n=59; 1, n=26; 2, n=29; 3, n=21; 4, n=2). **g**, Frequencies of on-target (A5) editing in input SCD HSPCs (n=3 for ABE7.10 and n=1 for ABE8e and Cas9) and in donor-derived cells in BM after transplantation (ABE7.10, n=11; ABE8e, n=3; Cas9, n=4). **h**, %HbF and i, %HbF versus on-target editing efficiency in hCD235a^+^ erythroblasts (UT and ABE7.10 alone, n=10; ABE7.10 −175 sgRNA, n=11; UT, ABE8e, and Cas9 only, n=5; ABE8e −175, n=3; Cas9, n=4). **j**, Percentages of BM–derived erythroid colonies with zero to four productive on-target edits (A5, not A3). **k**, Percentage of sickled cells in BM–derived human reticulocytes exposed to hypoxia (UT, n=10; ABE7.10, n=11). Bar charts show mean ± SD. For panels b, d, h, i, and k, *P* values were calculated using a linear regression model, adjusting for batch effects (panels b, h, and k). For panel c, *P* values were calculated using the Fisher’s combination test on the *P* values obtained by *t*-test. Each symbol (except for “input”) represents one mouse, with different symbols designating unique donors. UT, untreated.

To determine whether the γ-globin −175 A>G edit could be accomplished in bone marrow–repopulating HSCs, healthy donor CD34^+^ HSPCs were electroporated with ABE7.10 or ABE8e RNP and transplanted into non-obese diabetic/severe combined immunodeficiency/IL-2Rγ^−/−^/KitW41/W41 (NBSGW) mice that underwent necropsy after 16–18 weeks. Chimerism levels were similar for edited and unedited human donor–derived CD45^+^ (hCD45^+^) hematopoietic cells, CD3^+^ T cells, CD19^+^ B cells, CD33^+^ myeloid cells, and CD235a^+^ erythroid cells in the mouse bone marrow (Extended Data Fig. 9a–c). The on-target editing frequency in ABE7.10-edited input HSPCs was 28±2% at 3 days after electroporation and approximately 19% after transplantation in whole bone marrow cells, CD34^+^ HSPCs, and CD235^+^ erythroblasts, with no indels or bystander edits being detected (Fig. 5a, Extended Data Fig. 9d, Supplementary Table 7a). The on-target editing frequency with ABE8e was 59±7% in input HSPCs and approximately 39% after transplantation in whole bone marrow cells, HSPCs, and CD235^+^ erythroblasts, with no indels being detected (Fig. 5a, Extended Data Fig. 9d, Supplementary Table 7b). The CD235a^+^ erythroid populations generated after transplantation with unedited and edited donor HSCs were at similar stages of maturation (Extended Data Fig. 9e–h).

In human erythroid populations isolated from mouse recipient bone marrow, the F-cell fractions were 64±9% and 73±5% after editing with ABE7.10 and ABE8e, respectively, compared with approximately 6±2% in controls (Fig. 5b). The HbF fractions were 23±7% and 36±4% after editing with ABE7.10 and ABE8e, respectively, compared with 0.6% in controls. (Fig. 5c, Extended Data Fig. 9i). In individual mice, the %HbF correlated with the −175 A>G editing frequencies (Fig. 5d). Clonal analysis of human erythroid progenitors isolated from mouse bone marrow identified at least one productive −175 A>G edit (i.e., no A3 bystander edit, Figure 4d,e) in 66% of erythroid colonies derived from ABE7.10-edited HSPCs and in 85% of erythroid colonies derived from ABE8e-edited HSPCs (Fig. 5e). The %HbF in erythroid colonies correlated with the number of on-target γ-globin −175 A>G (A5) edits and tended to be lower when there were concomitant A3 bystander edits (Fig. 5f), as predicted by studies in HUDEP2 cells (Fig. 4d,e). However, the reduction in %HbF caused by the A3 bystander edit was outweighed by the increased on-target editing efficiency of ABE8e, which generated higher frequencies of HSPCs clones with one or more productive edits (A5 without A3) than ABE7.10.

**Extended Data Fig. 10 |:**
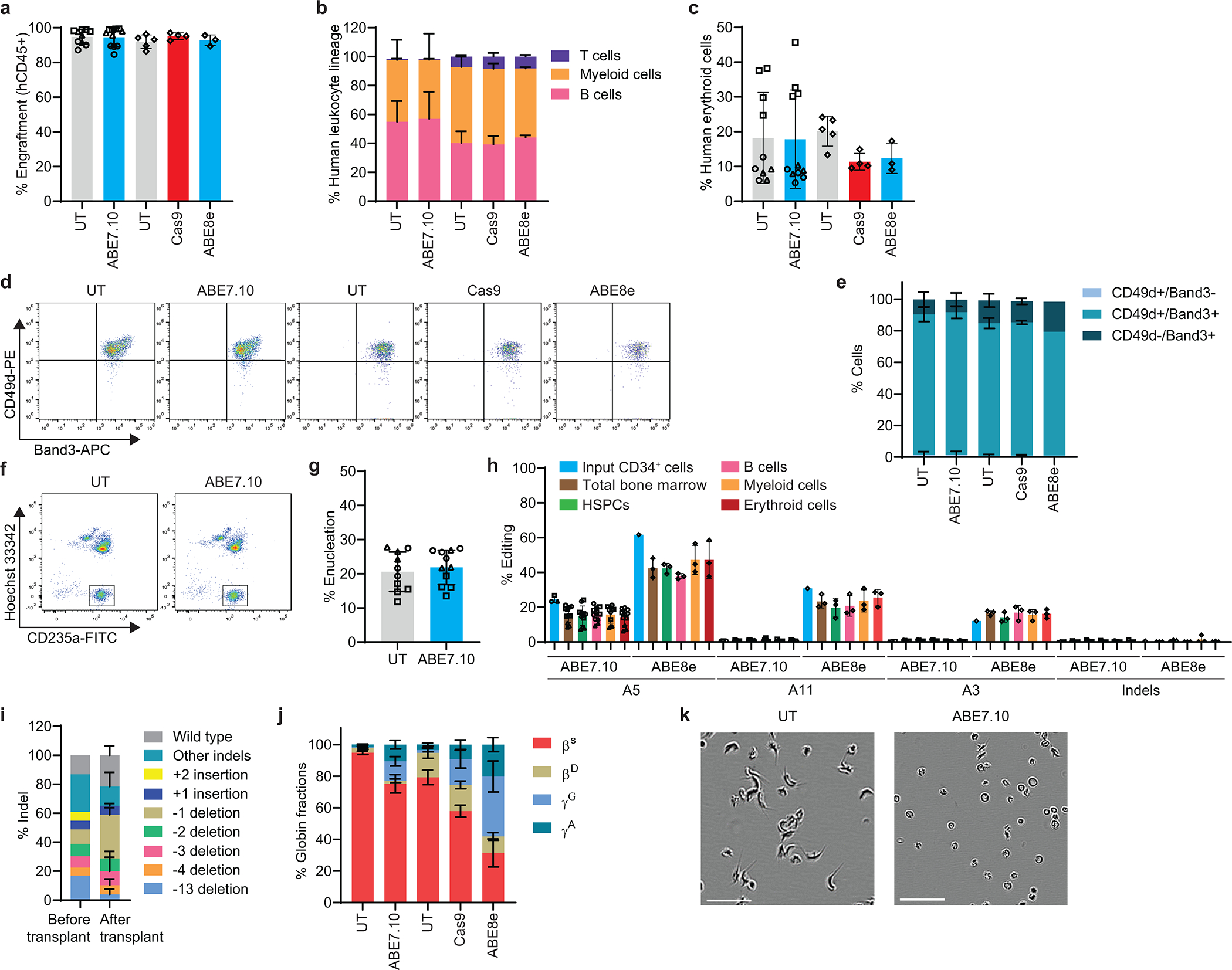
Durable editing in bone marrow–repopulating HSCs from SCD donors. **a,** Percentage of hCD45^+^ donor cells in mouse bone marrow at 16 weeks after transplantation. **b,** Percentages of B, myeloid, and T cells within the hCD45^+^ population from mouse bone marrow. **c,** Percentage of hCD235a^+^ erythroid cells in mouse bone marrow. **d,** Representative flow cytometry scatter plot of maturation markers in hCD235a^+^ erythroblasts in bone marrow. **e,** Summary of multiple experiments to assess erythroid cell maturation using gating strategy depicted in panel d. **f,** Representative flow cytometry scatter plot showing enucleated human reticulocytes in mouse bone marrow, distinguished by loss of staining with the DNA-binding dye Hoechst 33342 (gated). **g,** Percentage of enucleated hCD235^+^ erythroid cells (reticulocytes). **h,** On-target edits, bystander base edits, and indel frequencies in different bone marrow populations. **i,** Indel allele frequencies quantified in cells before transplantation (n=1 donor) and 16 weeks after transplantation (n=4 transplanted mice for Cas9 and n=3 for ABE8e). **j,** Percentages of β-like globin proteins in hCD235a^+^ erythroid cells recovered from mouse bone marrow. **k,** Human reticulocytes isolated from mouse bone marrow were incubated in 2% O_2_ for 8 h. The sickling assay was performed as two independent experiments from three donors (UT, n=10; ABE7.10, n=11). Representative micrographs show examples of sickled cells. Scale bar = 50 μM. For the experiments assessing ABE7.10, n=10 mice in the untreated samples and n=11 mice in the ABE7.10-treated samples, corresponding to three or four mice transplanted with cells from each of three different SCD donors. For the experiments assessing ABE8e and Cas9 nuclease, n=5 mice in the untreated samples, n=4 mice in the Cas9 treated samples, and n=3 mice in the ABE8e treated samples (all cells from one SCD donor). In panel e, n=2 mice in the ABE8e treated sample because one mouse did not yield sufficient CD235a^+^ cells for this analysis. All graphs show mean ± SD. Each symbol represents one mouse, with different symbols designating unique HSPC donors. UT, untreated; ns, not significant.

We compared the effects of treating SCD patient CD34^+^ HSPCs with ABE7.10 or ABE8e RNP to install γ-globin −175 or with Cas9 RNP to disrupt the γ-globin promoter BCL11A binding site. Sixteen weeks after transplantation into NBSGW mice, edited or control donor CD34^+^ cells produced similar chimerism levels of HSPCs, T cells, B cells, myeloid cells, and erythroid cells at similar developmental stages (Extended Data Fig. 10a–g). The on-target editing frequencies for Cas9, ABE7.10, and ABE8e were 85%, 25±2%, and 62%, respectively, in input cells and 75±5%, 16±5%, and 42±6%, respectively, post transplantation (Fig. 5g, Supplementary Table 8a,b). Bystander base-editing frequencies were similar in input and engrafted cells, with no indels detected (Extended Data Fig. 10h, Supplementary Table 8a,b). The distribution of indel alleles in Cas9-edited input cells differed from those observed after bone marrow engraftment, as was reported for cells edited at the *BCL11A* erythroid enhancer^31^ (Extended Data Fig. 10i).

The %HbF in donor-derived erythroid cells after editing with Cas9, ABE7.10, or ABE8e was 27±5%, 28±6%, and 62±8%, respectively (Fig. 5h, Extended Data Fig. 10j). In ABE7.10-edited and ABE8e-edited erythroblasts, the %HbF correlated linearly with the efficiency of on-target editing (Fig. 5i), and the %HbF per edit was approximately four times that in Cas9-edited erythroblasts (Fig. 5g,h). The frequencies of bone marrow–derived human erythroid colonies with at least one on-target edit were approximately 100%, 57%, and 77% for cells edited with Cas9, ABE7.10, and ABE8e, respectively (Fig. 5j). To test the therapeutic effects of base editing, we isolated SCD donor reticulocytes from bone marrow of mice that received ABE7.10-edited CD34+ cells and incubated them in 2% oxygen. The fraction of cells with hypoxia-induced sickling was 40±11%, compared to 73±4% in unedited control cells (Fig. 5k, Extended Data Fig. 10k). Thus, ABE installation of γ-globin −175 A>G in normal donor or SCD HSCs causes particularly potent induction of erythroid HbF in vivo.

### Off-target analysis.

**Fig. 6 |:**
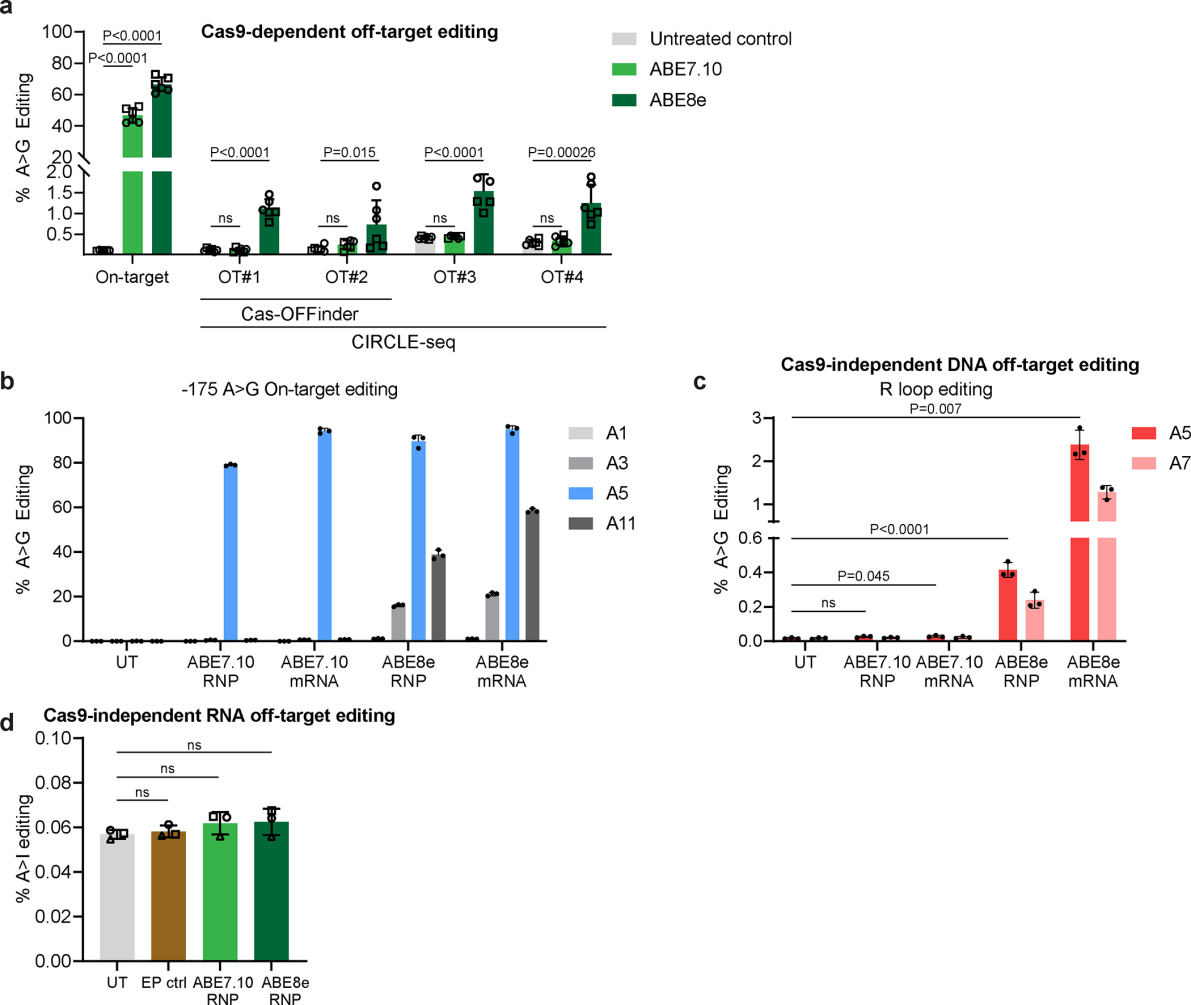
Off-target editing by ABE7.10 or ABE8e. **a,** CD34^+^ HSPCs from two different healthy donors indicated by different symbols were treated with ABE RNPs containing sgRNA −175. After six days, the potential off-target editing sites identified by CIRCLE-seq and/or Cas-OFFinder (212 sites) were evaluated by multiplex PCR and NGS. Significant off-target editing was observed in three sites and one additional site approached significance. Editing is calculated as the number of reads with an A•T-to-G•C transition mutation within the editing window (nucleotides 4–10 of the protospacer alignment) divided by the total number of reads. Three replicates for each of two different donors (n=6) were quantified for on-target and off-target editing. Off-target base editing was observed after treatment with ABE8e, but not ABE7.10. **b,c,** Orthogonal R-loop assay to measure Cas9-independent off-target editing with the editing reagents used in this study. HEK293T cells were transfected with plasmids encoding nuclease-inactive S. *aureus* Cas9 and sgRNA targeting an established genomic test site that leads to high off-target editing when held in an open R-loop. After 24 h, cells were electroporated with ABE8e or ABE7.10 (as mRNA or protein) and sgRNA −175, followed by PCR and NGS to detect base edits. **Panel b** shows frequencies of the on-target −175 A>G edit (n=3). Panel c shows the frequencies of Cas9-independent off-target editing at the R-loop (n=3). **d**, Cas9-independent editing of RNA. CD34^+^ HSPCs were edited as described for panel a, followed by RNA-seq analysis after six days. Bar graph shows the percentage of adenosine-to-inosine RNA modifications by electroporation control (EP ctrl), ABE7.10 RNP, and ABE8e RNP treated CD34^+^ HSPCs (n=3). The y-axis represents the percentage of A-to-I RNA editing. Each symbol represents an individual CD34+ cell donor. Bar graphs show mean ± SD with *P* values determined using a linear regression model after adjusting for the donor effect (panel a) and two-sample *t*-test (panel c and d, except UT vs ABE8e RNP in panel c, which was analyzed using a linear regression model). OT, off-target; UT, untreated; ns, not significant.

CIRCLE-seq identified 682 potential off-target sites for the Cas9-sgRNA RNP complex targeting γ-globin −175 (Supplementary Table 9). A parallel, *in silico* search using Cas-OFFinder identified 121 genomic sites with ≤3 mismatches to the sgRNA target sequence, 42 of which overlapped with sites identified by CIRCLE-seq (Supplementary Table 10). We treated CD34^+^ HSPCs with ABE7.10 or ABE8e RNP, then used multiplex-targeted DNA sequencing to measure the editing at 211 potential off-target sites nominated by CIRCLE-seq and/or Cas-OFFinder. We examined 41 of the 42 sites identified by both methods (one could not be amplified), 73 of the 79 sites identified by Cas-OFFinder only (six could not be amplified), and the top 97 sites identified by CIRCLE-seq only (three of the top 100 sites could not be amplified). In cells treated with ABE8e RNP, we identified low-frequency (<2%) off-target edits at four of 211 tested sites, which were located in intergenic DNA or in introns of *TMEM176B* or *PNPLA6* (Fig. 6a, Supplementary Table 11). All four sites were identified by CIRCLE-seq, and two were also identified by Cas-OFFinder. No off-target editing was observed after treatment with ABE7.10 RNP.

We used an R-loop assay^44^ to investigate the potential for Cas9-independent off-target base editing of genomic DNA by ABE7.10 or ABE8e. This assay uses catalytically inactive Staphylococcus aureus Cas9 (saCas9) and sgRNA to sensitize a small region of genomic DNA to Cas9-independent ABE deamination by holding it in an unwound single-stranded conformation. We transfected HEK293 cells with plasmids encoding nuclease-inactive saCas9 and sgRNA targeting a validated test site^41,44^. One day later, cells were electroporated with γ-globin −175 sgRNA and ABE7.10 or ABE8e delivered as mRNA or RNP. Efficient on-target editing (79–96%) was detected in all samples (Fig. 6b). Cas9-independent off-target DNA editing frequencies at the R-loop with ABE7.10 delivered as mRNA or RNP were 0.029±0.005% and 0.026±0.005%, respectively, compared with 0.017% in non-electroporated controls (*P* = 0.045 for mRNA and 0.065 for RNP). Higher levels were observed with ABE8e mRNA and protein: 2.4±0.3% (*P* = 0.007) and 0.4±0.04% (*P* < 0.0001), respectively (Fig. 6c). These results are consistent with the higher activity of ABE8e relative to ABE7.10 and with more prolonged ABE exposure after mRNA delivery^41^.

To measure Cas9-independent off-target editing of mRNA, we electroporated CD34^+^ cells from three different normal donors with ABE7.10 or ABE8e RNP, followed by high-depth RNA-seq (4.4–6.7×10^7^ reads) after six days. The rates of A-to-I conversion were similar in edited and unedited cells (Fig. 6d), consistent with previous reports that off-target RNA editing was not detected after adenine base editing in an erythroid cell line or HSPCs^17,21,^

## Discussion

New approaches to inducing HbF for treating β-hemoglobinopathies are fueled by the development of genome engineering technologies and insights into globin gene regulation^3^. The field is evolving rapidly, and the best strategy is not known. We compared the use of ABEs to generate three HPFH γ-globin promoter and two nuclease approaches that inhibit the expression of the BCL11A repressor or interfere with its binding to the γ-globin promoter. Our study informs therapeutic strategies for HbF induction and provides new insights into mechanisms of globin gene transcription.

In contrast to Cas9 editing, ABE generation of −175 A>G was not associated with TP53 activation. Base editing may be particularly advantageous for modifying the duplicated γ-globin genes because simultaneous nuclease targeting of their nearly identical promoters will produce two DSBs and can result in a 4.9-kb deletion. Two DSBs per gene cluster could enhance the DNA damage response and compromise HSCs to a greater extent than nuclease editing at a single genomic site. In vitro differentiation of modified CD34^+^ cells showed that the −175 A>G γ-globin variant was approximately two to three times more potent than Cas9 indels for inducing HbF (Fig. 1e). In reticulocytes generated in vivo from −175 A>G–modified SCD CD34^+^ cells, the %HbF per %edit was approximately four times that of cells edited to disrupt the γ-globin promoter *BCL11A* motif^8^ or of the BCL11A erythroid enhancer^31^. Moreover, the −175 A>G variant resulted in more consistent levels of HbF across RBC clones compared to those with Cas9-generated indels.

Beginning shortly before birth, the BCL11A and ZBTB7A repressors bind distinct motifs in the γ-globin promoter to silence gene expression additively^34,45–47^. Disrupting either motif at a single γ-globin gene, in adult-type erythroid precursors raises HbF levels to approximately 50%, whereas disrupting both motifs results in nearly 100% HbF^45^. Installing −175 A>G resulted in 80–90% HbF. Thus, one ABE edit at γ-globin −175 replaces two separate genetic manipulations that interfere with repression by BCL11A and ZBTZ7A. These findings are consistent with observations that individuals with γ-globin −175 A>G HPFH express higher levels of HbF than individuals with HPFH alleles that disrupt BCL11A or ZBTB7A binding^7,48,49^. The −175 A>G variant induces γ-globin transcription by assembling a promoter TAL1-GATA1-LDB1-LMO2 complex that mediates interactions with the upstream LCR^29,39,50^ (Extended Data Fig. 6f). Similarly, recruitment of the LDB1 self-association domain to the γ-globin promoter via a DNA-localizing artificial zinc finger stimulated LCR contacts and activated transcription to 85% of total β-like globin synthesis^51^.

Surprisingly, 1-, 2- or 3-bp deletions that disrupted the γ-globin promoter BCL11A binding motif (TGACCA) induced HbF less effectively than did 4- or 13-bp deletions in the same region. The smaller deletions may support the binding of a different repressor protein or interfere with the recruitment of a transcriptional activator, possibly by creating unfavorable base-pair and/or nucleosome spacing. In contrast to these findings, all high-frequency indels at the *BCL11A* erythroid enhancer caused similar levels of HbF induction on average, although the magnitude varied considerably between different erythroid clones that were homozygous for the same indel. These indels disrupt a functional GATA motif in the multi-component enhancer and do not eliminate enhancer activity or erythroid *BCL11A* expression fully^32^. In this case, variable HbF outcomes may result from stochastic effects of residual gene expression in different erythroid clones. Further studies are required to address these possibilities.

Our data indicate that ABE7.10 and ABE8e have different advantages for editing the −175 A>G target. ABE8e yielded higher editing efficiency and higher HbF induction, but ABE7.10 caused fewer bystander and off-target edits. Whereas most prior studies expressed base editors in CD34^+^ cells by delivering mRNA^19,38,52^, we achieved therapeutic levels of editing by delivering ABE RNP, which reduced the potential for Cas9-independent off-target editing of DNA by ABE8e. Through further protein engineering, it should be possible to further enhance the specificity and activity of base editing at the γ-globin −175 target site^19,41,53–55^.

Genetic induction of γ-globin/HbF represents a powerful generalized approach to treating SCD and most forms of β-thalassemia, which can be caused by hundreds of different *HBB* mutations^1–3^. Approximately 30% HbF distributed equally in all RBCs alleviates most or all SCD symptoms, although higher levels are preferable because therapeutic thresholds vary according to organ pathology and patient age^3,56^. Homogeneous, HbF induction is essential to inhibit sickle hemoglobin polymerization in all RBCs. Optimal therapeutic efficacy may be best achieved by efficient ABE installation of γ-globin −175 A>G, which results in stronger and more consistent HbF induction than that obtained with Cas9 nuclease editing. More broadly, our findings demonstrate that uncontrolled mixtures of Cas9 indels may contain mutant alleles with unanticipated, unfavorable biological consequences that can be avoided with more precise base editors.

## Methods

All research complies with our Institution Biosafety Committee and the St. Jude Institutional Animal Care and Use Committee (IACUC protocol 579) and performed according to relevant ethical regulations.

### Human subjects research.

Plerixafor-mobilized and bone marrow-derived CD34^+^ cells from patients with SCD were collected according to the protocol Peripheral Blood Stem Cell Collection for Sickle Cell Disease Patients (www.clinicaltrials.gov identifier #NCT03226691), which was approved by the human subject research institutional review boards at the National Institutes of Health and St. Jude Children’s Research Hospital. All patients provided informed consent.

### Animal care.

Mice were housed and handled in strict accordance with the recommendations in the Guide for the Care and Use of Laboratory Animals of the National Institutes of Health. Animal experiments were carried out in accordance with a protocol (Genetic Tools for the Study of Hematopoiesis) approved by the Institutional Animal Care and Use Committee of St. Jude Children’s Research Hospital.

### Genome-editing reagents.

Modified synthetic sgRNAs containing 2′-O-methyl modified nucleotides in the first three and last three nucleotides and 3′ phosphorothioate bonds between the first three and last two nucleotides were purchased from Synthego or Biospring. Sequences of sgRNA used in this study are described in Supplementary Table 12. ABE7.10-SpCas9^13^ was used to generate −198 A>G and −113 A>G and ABE7.10-NG^57^ was used to generate −175 A>G. ABE7.10 and ABE7.10-NG were purchased as custom products from Aldevron and delivered at stock concentrations of 94 μM and 45.9 μM respectively. Cas9–3xNLS was manufactured by the St. Jude Protein Core (Supplementary Note 1)^8,31^.

ABE8e-SpCas9-NG^58^ cDNA was cloned into the protein expression plasmid pD881-SR (Atum, cat. no. FPB-27E-269), which was then used to transform BL21 Star DE3 competent cells (Thermo Fisher, cat. no. C601003). Colonies were picked for overnight growth in terrific broth (TB) + 25 μg/mL kanamycin at 37 °C. Next day, 2 L of pre-warmed TB were inoculated with overnight culture at a starting OD600 of 0.05. Cells were shaken at 37 °C for approximately 2.5 h until the OD600 was ~1.5. Cultures were cold shocked in an ice-water slurry for 1 h, after which L-rhamnose was added to a final concentration of 0.8% to induce protein expression. Cultures were then incubated at 18 °C with shaking for 24 h to enable the cells to express protein. After induction, cells were collected by centrifugation and the pellet was flash-frozen in liquid nitrogen and stored at −80 °C. Next day, the cells were resuspended in 30 mL of cold lysis buffer (1 M NaCl, 100 mM Tris-HCl pH 7.0, 5 mM TCEP, 20% glycerol, with 5 tablets of complete, EDTA-free protease inhibitor cocktail (Millipore Sigma, cat. no. 4693132001). Cells were passed three times through a homogenizer (Avestin Emulsiflex-C3) at ~18,000 psi to lyse them. Cell debris was collected by centrifugation at 20,000 rcf for 20 min at 4 °C. The supernatant was collected and spiked with 40 mM imidazole. This was followed by a 1-h incubation at 4 °C with 1 mL of Ni-NTA resin slurry (G Bioscience cat. no. 786–940, prewashed once with lysis buffer). Protein-bound resin was washed twice with 12 mL of lysis buffer in a gravity column at 4 °C. Protein was eluted in 3 mL of elution buffer (300 mM imidazole, 500 mM NaCl, 100 mM Tris-HCl pH 7.0, 5 mM TCEP, 10% glycerol). Eluted protein was diluted in 40 mL of low-salt buffer (100 mM Tris-HCl, pH 7.0, 1 mM TCEP, 20% glycerol) just before loading into a 50 mL Akta Superloop for ion exchange purification on an Akta Pure25 FPLC system. Ion-exchange chromatography was conducted on a 5 mL GE Healthcare HiTrap SP HP pre-packed column (cat. no. 17115201). After the column was washed with low-salt buffer, the diluted protein eluate was allowed to flow through the column to enable the protein to bind. The column was then washed in 15 mL of low-salt buffer before being subjected to a gradient increasing to a maximum of 80% high-salt buffer (1 M NaCl, 100 mM Tris-HCl, pH 7.0, 5 mM TCEP, 20% glycerol) over the course of 50 mL, at a flow rate of 5 mL/min. Fractions of 1 mL were collected during this ramp to high-salt buffer. Peaks were assessed by SDS-PAGE to identify fractions containing the desired protein, which were concentrated first with an Amicon Ultra 15-mL centrifugal filter (100-kDa cutoff, cat. no. UFC910024) and then with a 0.5-mL 100-kDa cutoff Pierce concentrator (cat. no. 88503). The ABE8e-SpCas9-NG protein was quantified using a BCA assay (Thermo Fisher, cat. no. 23227) and concentrated to a stock concentration of 98.77 μM.

### Isolation and maintenance of CD34+ hematopoietic stem and progenitor cells (HSPCs).

Circulating G-CSF–mobilized human mononuclear cells were obtained from de-identified healthy adult donors (Key Biologics, StemExpress). Plerixafor-mobilized mononuclear cells were isolated from patients with SCD. CD34+ cells were enriched by immunomagnetic bead selection using an AutoMACS instrument (Miltenyi Biotec). CD34^+^ cells were maintained in CD34^+^ expansion medium: X-VIVO 10 (Lonza, BEBP02–055Q) medium supplemented with 100 ng/μL recombinant human SCF (R&D systems, 255-SC/CF), 100 ng/μL recombinant human TPO (R&D systems, 288-TP/CF), and 100 ng/μL recombinant Flt-3 ligand (R&D systems, 308-FK/CF), with a seeding density of 0.5–1 × 10^6^ cells/mL.

### Genome editing of CD34^+^ HSPCs.

Electroporation was performed with either a Lonza 4D Nucleofector (Lonza, V4SP-3096) or a MaxCyte GTx electroporator, used in accordance with the manufacturer’s instructions. CD34^+^ HSPCs were thawed 24–48 h before electroporation. For 20-μL Nucleocuvette Strips, the RNP complex was prepared by mixing ABE7.10 or ABE8e protein (5 μM or 8 μM, respectively) and sgRNA (15 μM or 28 μM, respectively) and incubating the mixture for 20 min at room temperature before adding it to cells. For base editing, the electroporation conditions were optimized using a design of experiments (DoE) statistical strategy (Extended Data Fig. 8a). The editor, gRNA, and cell concentrations were evaluated to maximize editing efficiency and cell recovery by using a central composite, response surface design. Least-squares fit effect screening was performed to determine the optimal concentration conditions using the JMP statistical analysis package (JMP^®^, Version 16.2.0., SAS Institute Inc., Cary, NC). Two hundred thousand to 2 million HSPCs resuspended in P3 solution were mixed with RNP and transferred to a cuvette for electroporation with program DS-130. For MaxCyte electroporations, 5 million HSPCs resuspended in MaxCyte buffer solution were mixed with RNP and transferred to an OC-100 cartridge for electroporation with program HSC3.

For Cas9 editing, the optimal concentration of RNP (with the Cas9 component at a 1:3 molar ration with sgRNA) was determined by dose titration (Extended Data Fig. 5). For most studies using Cas9–3xNLS, the nuclease (5 μM) was incubated for 20 min at room temperature with sgRNAs (15 μM) targeting the γ-globin promoters or the *BCL11A* erythroid enhancer (Supplementary Table 12). It was then added to CD34^+^ cells and electroporated using the Lonza Nucleofector, as described for ABE proteins. For 100-μL cuvette electroporation, the RNP complex was made by mixing 25 μM ABE7.10 protein and 75 μM sgRNAs with 2.5–5 million CD34^+^ HSPCs. For studies comparing Cas9 and ABE8e in SCD CD34^+^ cells (Fig. 5), transfection was performed using the GTx MaxCyte electroporator (program HSC3).

Electroporated cells were recovered in X-VIVO 10 medium including cytokines. Genomic DNA was extracted on culture days 3 and 6 by using Qiagen buffer then analyzed by NGS for editing efficiency.

### Next-generation sequencing library preparation and analysis.

Targeted amplicons were generated using gene-specific primers with partial Illumina adapter overhangs (Supplementary Table 14) and sequenced as follows^59^.

Cell pellets (0.1 million cells) were lysed and the extracted genomic DNA was used as a template in PCR #1 to amplify the target site and add Illumina adapters. Amplicons were indexed in PCR #2 and pooled for sequencing. 10% PhiX Sequencing Control V3 (Illumina) was added to the pooled amplicon library before the sample was run on a MiSeq Sequencer System (Illumina) to generate paired 2 × 250-bp reads. Samples were demultiplexed using the index sequences, fastq files were generated, and NGS analysis were performed using CRIS.py analysis (see Supplemental Table 15 for program parameters)^60^.

### Erythroid cell culture.

CD34+ HSPCs were differentiated into erythroid cells in vitro by using a three-phase protocol as follows^8,11,61^. For phase 1 (days 1–7), cells were maintained at a density of 1.0–5.0 × 10^5^ cell/mL in Iscove’s modified Dulbecco’s medium (IMDM; Thermo Fisher Scientific, 12440061) supplemented with 2% human blood type AB plasma (SeraCare, 1810–0001), 3% human AB serum (Atlanta Biologicals, S40110) 1% penicillin/streptomycin (Gibco, #15140122), 3 units/mL heparin (Sagent Pharmaceuticals, NDC 25021–401-02), 3 units/mL EPO (Amgen, EPOGEN NDC 55513–144-01), 200 μg/mL holo-transferrin (Millipore Sigma, T0665, 10 ng/mL recombinant human SCF (R&D systems, 255-SC/CF), and 1 ng/mL recombinant human interleukin IL-3 (R&D systems, 203-IL/CF). For phase 2 (days 8–14), cells were maintained at a density of 1.0–5.0 × 10^5^ cell/mL in phase 1 medium without IL-3. For phase 3 (days 15–21), cells were maintained at a density of 1.0–2.0 × 10^6^ cell/mL in phase 2 medium without SCF and with the holo-transferrin concentration increased to 1 mg/mL.

Erythroid maturation during in vitro differentiation was measured by immuno-flow cytometry for the cell surface markers CD235a, CD49d and Band3 (Supplementary Table 13). For erythroid enucleation rate determination and F-cell population analysis, 1.0−2.0 × 10^5^ CD34^+^ HSPC-derived erythroid cells were incubated with Hoechst 33342 for 20 min at 37°C, fixed with 0.05% glutaraldehyde (Millipore Sigma, G5882), and permeabilized with 0.1% Triton X-100 (Millipore Sigma, 93443). Subsequently, cells were stained with antibodies against human CD235a and HbF and analyzed by flow cytometry (Supplementary Table 13).

### Generation and analysis of burst-forming unit erythroid (BFU-E) colonies.

Edited or untreated CD34^+^ HSPCs (500,000 cells) were incubated for 3 days in Phase 1 medium then seeded into 1 mL of metho-cult medium containing erythroid cytokines (Stem Cell Technologies, H4433) in a 35-mm tissue culture dish (Corning). After 14 days, individual colonies were collected with a micropipette, resuspended in 15 μL of hemolysis buffer (Helena Laboratories), and centrifuged at 4000 rcf for 15 min. Ten microliters of supernatant was used for quantifying the HbF by ion-exchange HPLC. Five microliters of DNA extraction buffer was added to the remaining 4-μL pellet to prepare genomic DNA and enable genotype analysis.

### Xenotransplantation of gene-edited CD34+ HSPCs into NOD.Cg-KitW-41J Tyr+ Prkdcscid Il2rgtm1Wjl/ThomJ (NBSGW) mice.

NBSGW mice were purchased from The Jackson Laboratory (stock no. 026622). Base-edited, Cas9–edited, or untreated CD34+ cells were administered at a dose of 2.0–5.0 × 10^5^ cells per mouse by tail-vein injection into (3–6 mice per condition) female mice aged 8–12 weeks. Chimerism post transplantation was evaluated at 16–18 weeks in the bone marrow at the time of euthanasia. Cell lineage composition was determined in the bone marrow by using human-specific antibodies (Supplementary Table 13), and lineages were sorted using a FACSAria III cell sorter (BD Biosciences). CD34+ HSPCs and CD235a+ erythroblasts were isolated with magnetic beads, using the human specific CD34 MicroBead Kit UltraPure (Miltenyi Biotec, 130–100-453) and human CD235a (glycophorin A) MicroBeads (Miltenyi Biotec, 130–050-501).

### HbF quantification.

Analytical high-performance liquid chromatography (HPLC) quantification of hemoglobin tetramers and individual globin chains was performed using ion-exchange and reverse-phase columns on a Prominence HPLC System (Shimadzu Corporation). Proteins eluted from the column were identified at 220 and 415 nm with a diode array detector. The relative amounts of hemoglobins or individual globin chains were calculated from the area under the 418-nm peak and normalized based on the dimethyl sulfoxide control. The percentage of HbF was calculated from ion-exchange HPLC as follows: %HbF = [HbF/(HbA + HbF)] × 100. The percentage of γ-globin hemoglobin subunits was calculated from reverse-phase HPLC as follows: % γ-globin = [(Gγ-chain + Aγ-chain)/β-like chains (β + Gγ + Aγ)] × 100. To measure the %HbF induced per edit in bulk populations (Fig. 1e), we first calculated the total editing efficiency and %HbF for each editing condition and unedited control. For each donor, we averaged the %HbF measured in any replicates of the untreated (UT) samples to serve as a normalization value, since baseline HbF expression tends to vary between individuals. Then, we normalized the %HbF measurement of each edited sample by subtracting the %HbF average that was calculated for UT samples of the same donor. Lastly, we divided the normalized %HbF by the editing efficiency (% edit) of the same sample to obtain %HbF/% editing (Supplementary Table 1, Fig. 1e).

### HUDEP cell expansion and differentiation.

HUDEP1 and HUDEP2 cells were obtained from R. Kurita and Y. Nakamura (RIKEN BioResource Center). HUDEP cells were expanded in culture medium consisting of SFEM (Stem Cell Technologies, 09650) supplemented with 50 ng/mL recombinant human SCF, 3 IU/mL EPO, 1 μg/mL doxycycline (Sigma Aldrich, D9891), 0.4 μg/mL dexamethasone (Sigma Aldrich, D4902), and 1% penicillin-streptomycin solution. HUDEP2 cells were differentiated using a two-phase protocol. In phase 1 (days 1–3), cells were maintained in IMDM with 2% fetal bovine serum, 2% human blood type AB plasma, 1% penicillin/streptomycin, 3 units/mL heparin, 10 μg/mL insulin (Sigma, I9278), 3 units/mL EPO, 1mg/mL holo-transferrin, 50 ng/mL SCF and 1 μg/mL doxycycline. In phase 2 (days 5–10), cells were maintained in phase 1 medium without SCF.

### Generation of HUDEP2 clones.

Wild-type HUDEP2 cells and hemizygous HUDEP2 cells harboring a single allele of β-like globin genes opposite a large deletion on the homologous chromosome (HUDEP2^Δεγδβ^)^34^ were electroporated with ABE7.10 and or ABE8e RNP complexed with sgRNA targeting −175 A>G, using a Lonza 4D nucleofector system with the DS-150 program. Edited wild-type HUDEP2 cells and hemizygous HUDEP2 cells were sorted into 96-well round-bottom plates (Corning) at one cell per well in 100 μL of expansion medium, using a FACSAria III cell sorter (BD Biosciences) or an SH800 sorter (Sony Biotechnologies). The cells were switched to 24-well plates (Corning) 14 days later. After an additional 4 days of expansion, cells were harvested for genotyping analysis. Likewise, HUDEP2^Δεγδβ^ cells were electroporated with sgRNA (−187/189) and clones were picked that had A>G edits at positions −187 and −189 relative to the transcription start site to generate *HBG1*/2^ΔGATA^ clones (Fig. 2a). Subsequently, *HBG1*/2^ΔGATA^ cells were electroporated with sgRNA (−175) to generate clones containing all three edits (*HBG1/2*^−175G/ΔGATA^). HUDEP2 cells that harbored a single copy of the γ-globin gene (HUDEP2^ΔεγδβGγ/Aγ^ cells) were electroporated with RNP complex consisting of Cas9 and sgRNA targeting the γ-globin promoter to generate clones with a spectrum of γ-globin promoter indels (Fig. 3h). All of the clones were differentiated using the two-phase culture protocol, and the %HbF was measured by ion-exchange and reverse-phase HPLC.

### In vitro RBC sickling assay.

CD34+ cell–derived erythroid cells on day 21 in culture and MACS purified CD235a+ cells from bone marrow were incubated with Hoechst 33342 for 20 min at 37°C, and the Hoechst-negative population was sorted using an SH800 sorter (Sony Biotechnologies). Sorted cells (0.5–1.0 × 10^5^ cells) were recovered in phase 3 erythroid differentiation (ED) medium for 24 h and seeded in 12-well or 96-well plates with 1.0 mL and 100 μL of phase 3 ED medium respectively under hypoxic conditions (2% O_2_) for 24 h. The IncuCyte^®^ S3 Live-Cell Analysis System (Sartorius) with a 20× objective was used to monitor cell sickling, with images being captured every 20–30 min. The percentage of sickling was measured after 8 h in hypoxia by manual counting of sickled cells versus normal cells based on morphology. For each condition, at least 300 cells were counted in three different microscopic fields by a blinded observer.

### Reverse transcription digital droplet PCR.

The concentration of the extracted RNA was measured using a NanoDrop system (Thermo Fisher Scientific). One-step reverse transcription digital droplet PCR (RT-ddPCR) was used to determine the p21 mRNA expression change. Ribonuclease P/MRP subunit p30 (RPP30) was used as an internal control. For each extract, 3 ng of RNA was mixed with reverse transcriptase, 300 mM DTT, and Supermix from a One-Step RT-ddPCR Advanced Kit for Probes (Bio-Rad, Hercules, CA), p21 primers/probe (Bio-Rad, 10031252; Assay ID: dHsaCPE5052298), and RPP30 primers/probe (Bio-Rad, 10031255; Assay ID: dHsaCPE5038241), according to the manufacturer’s protocol. After droplets were formed with an Automated Droplet Generator (Bio-Rad), PCR was performed as follows: first step at 50 °C for 60 min; second step at 95 °C for 10 min; third step at 95 °C for 30 s and then at 55 °C for 1 min (40 cycles); and fourth step at 98 °C for 10 min. Droplets were read using QX200^™^ (Bio-Rad), and data were analyzed using QuantaSoft (Bio-Rad).

### qPCR detection of 4.9-kb deletion alleles.

Quantification of 4.9-kb deletions by qPCR performed^11^ using a primer and probe set designed to detect amplification of an *HBG1* promoter–specific sequence (Supplementary Table 14). TaqMan qPCR was performed on genomic DNA samples from untreated and gene-edited CD34+ cells using Universal TaqMan Mix (Thermo Fisher Scientific), with quantification of triplicates for each sample. The results were compared to those obtained with control HUDEP-2 clones containing 0, 1, or 2 4.9-kb–deleted alleles. ΔΔCt values were calculated based on the amplification of RNaseP gene (*RPPH1*) (Thermo Fisher Scientific) as a copy number reference.

### COS-7 cell transfections and nuclear extraction.

COS-7 cells were cultured in Dulbecco’s Modified Eagle Medium (DMEM, Gibco) supplemented with 10% fetal bovine serum (FBS, Interpath), and 1% Penicillin-Streptomycin-Glutamine (PSG, Gibco). Cells were lifted for passaging by incubation in 0.05% Trypsin-EDTA (Gibco) at 37°C for 5 minutes. COS-7 cells were transfected with 5 μg of mammalian pcDNA3 expression plasmid (Invitrogen) containing FLAG-BCL11A zinc fingers 4–6^62^ or empty vector using FuGENE 6 (Promega). Transfected cells were incubated at 37 °C for 48 h before harvest. Cells were washed in PBS before harvesting for nuclear extraction by scraping and centrifugation. Cells were resuspended in 10× packed cell volume of hypotonic lysis buffer (10 mM HEPES (pH 7.9), 1.5 mM MgCl_2_, 10 mM KCl, 5 mM dithiothreitol, 1 mM phenylmethylsulfonyl fluoride, 10 μg/ml aprotinin, 10 μg/μl leupeptin) and incubated on ice for 10 min. The suspension was thoroughly vortexed to aid lysis before pelleting in a quick-spin centrifuge and discarding the supernatant. The pellet was resuspended in 2 to 3× packed cell volume of extraction buffer (20 mM HEPES (pH 7.9), 1.5 mM MgCl_2_, 0.42 M NaCl, 0.2 mM EDTA, 5 mM dithiothreitol, 1 mM phenylmethylsulfonyl fluoride, 10 μg/ml aprotinin, 10 mg/μl leupeptin, and 25% glycerol) and incubated on ice for 20–30 min. The suspension was centrifuged at 16,000 rcf for 3 min at 4 °C and the supernatant recovered.

### The Electrophoretic Mobility Shift Assay (EMSA)

The sense strand (Supplementary Table 14) for each probe was labelled with ^32^P from γ-^32^P ATP (Perkin Elmer), using T4 PNK (NEB), then annealed to the antisense strand (Supplementary Table 14) by slow cooling from 100 °C to room temperature. Annealed probes were then purified using Quick Spin Columns for Radiolabelled DNA Purification (Roche). To assess BCL11A binding, nuclear proteins from COS-7 cells overexpressing FLAG-BCL11A zinc finders 4–6 or empty vector were complexed with labelled probes made up in gel shift buffer (10 mM HEPES (pH 7.8), 50 mM KCl, 5 mM MgCl2, 1 mM EDTA, 50 μg/mL poly(dI-dC), 1 mM DTT, 100 μg/mL bovine serum albumin, 5% Glycerol) with or without antibody to FLAG (Sigma-Aldrich, F1804). Samples were loaded on 6% native polyacrylamide gel in TBE buffer (45 mM Tris, 45 mM boric acid and 1 mM EDTA). Electrophoresis was performed at 4 °C and 250 V for 1 h and 40 min, and the gel was vacuum dried before exposure to a FUJIFILM BAS Cassette2 phosphor screen overnight. Imaging was performed using a GE Typhoon FLA 9500 scanner.

### CUT&RUN assay.

CUT&RUN assays were performed to identify the occupancy of transcription factors as follows. Approximately 5.0 × 10^5^ cells were collected for each sample. Centrifuge 3 mins 600 rcf at room temperature and withdraw culture medium. Resuspend in 1 ml of wash buffer at RT by gently pipetting and Centrifuge for 3 min at 600 rcf at RT and withdraw the buffer, repeat this step for one more time. Resuspend in 1 ml of wash buffer at RT by gently pipetting, add 20ul of the Concanavalin A–coated magnetic beads (Bangs Laboratories, cat. no. BP531), rotate for 10 min at RT. Place the tubes on the magnet stand for 2–5 min until the solution turns clear, then remove and discard the liquid. Dilute antibodies with antibody buffer at 1:100, resuspend the beads in 200 μl of the antibody buffer (per sample). Transfer the liquid contains beads to 500 μl tubes, Place the tubes on the tube rotator at 4 °C for ~2 h. Remove the liquid from the caps and the sides of the tubes with a quick spin on a microcentrifuge (<100 rcf, RT, 1 s). Place the tube on the magnet stand until the solution turns clear and remove all the liquid. Add 1 ml of digitonin wash buffer, mix by pipetting, and transfer the liquid to 1.5ml tube, place the tubes on the magnet stand until the solution turns clear, then remove and discard the liquid. Repeat these steps for one more time. Resuspend beads in the pA-MN to a final concentration of ~700 ng/ml at volume of 200 μl, then transfer the solution into a 500 μl tube. Place on the tube rotator at 4 °C for ~1 h. Place the tube on the magnet stand until the solution turns clear and remove all the liquid. Add 1 ml of digitonin wash buffer, mix by pipetting, and transfer the liquid to 1.5ml tube, place the tubes on the magnet stand until the solution turns clear, then remove and discard the liquid. Repeat these steps for two more times. Remove the liquid from the cap and sides of the tube with a quick pulse (<100 rcf, ~22 °C, ~1 s) on a microcentrifuge. Add 150 μl of digitonin buffer to resuspend the beads. Transfer to a 1.5ml EP tube, insert the tubes into 1.5-ml wells of a heater block setting to 0 °C. Remove each tube from the block, mix in 3 μl of 100 mM CaCl2 with gentle pipetting and immediately replace the tube in the 0 °C block. Incubate at 0 °C for 1h. Add 150 μl of 2× stop buffer and mix by gentle pipetting. Incubate the tube for 10 min at 37 °C to release CUT&RUN fragments from the insoluble nuclear chromatin. Centrifuge for 5 min at 4 °C at 16,000 rcf and place the tube on a magnet stand. Centrifuge for 5 min at 4 °C at 16,000 rcf and place the tube on a magnet stand. Collect supernatant and extract DNA by regular Phenol-Chloroform method. The antibodies used for CUT&RUN are listed in Supplementary Table 13. Library construction for DNA sequencing was performed using a NEBNext Ultra II DNA Library Prep Kit from New England BioLabs (NEB) (E7645S). Indexed samples were analyzed by paired end NGS, using an Illumina NextSeq Mid-Output (150 cycles) Kit. FASTQ files were mapped to hg19 by using BWA-MEM (version 0.7.16a). Reads that could not be uniquely mapped to the human genome were removed by SAMtools (version 0.17; samtools view -q 1). Peaks were called using MACS2 (version 2.1.1). Data were normalized using S3norm^63^.

### Micro Capture-C.

Micro Capture-C^63^ was performed as follows 1–2 × 10^7^ cells were fixed for 10 min at room temperature with formaldehyde (Sigma # 252549) at a final concentration of 2% and quenched with 1 M glycine (Sigma # G7126). After centrifugation, the cell pellet was washed and reconstituted in 1 mL of PBS and permeabilized with 0.005% digitonin (Sigma # D141) for 5–10 min. Permeabilized cells were collected by centrifugation at 500 rcf for 5 min and resuspended in low-calcium micrococcal nuclease (MNase) buffer (50 mM Tris-HCl pH 7.5, 1 mM CaCl2). Cells were digested with MNase (NEB # M0247) (7.5 to 50 Kunitz Units) in a reaction volume of 800 μL containing 4 × 10^6^ cells at 37 °C for 1 h on an Eppendorf Thermomixer at 550 rpm. The reaction was quenched by adding ethylene glycol-bis(2-aminoethylether)-N,N,N′,N′-tetra acetic acid (EGTA) (bioWORLD # 405200085) to a final concentration of 5 mM. A 200 μL aliquot was removed as a control to test the digestion efficiency. The reaction mixture was then centrifuged for 5 min at 300 rcf, and the digestion buffer was discarded. The cells were resuspended in DNA ligase buffer (Thermo Fisher # EL0013) with 10 mM dNTPs (NEB # N0447L), 5 mM EGTA, 200 U/mL T4 polynucleotide kinase PNK (NEB # M0201L), 100 U/mL DNA polymerase I large (Klenow) fragment (NEB # M0210L), and 300 U/mL T4 DNA ligase (Thermo Fisher # EL0013). The reaction mixture was shaken horizontally on an Eppendorf Thermomixer at 550 rpm (shaking for 15 s then pausing for 30 s) for 2 h at 37 °C then for 8 h at 20 °C. The chromatin was reverse crosslinked with proteinase K at 65 °C (for >4 h), and the DNA was extracted using a Qiagen DNeasy Kit (# 69506). Next, 1–10 μg of DNA from the MNase 3C libraries was sonicated with a Covaris E220 Focused Ultrasonicator (Duty cycle, 10%; intensity, 5; cells per burst, 200; time, 200–300 s). Probes used for capture are listed in Supplementary Table 14. Data were analyzed with a custom analysis pipeline specifically developed for MCC data analysis^63^. Bait sequences were extracted using MCC_BLATfa.pl. Paired-end reads were mapped to the bait sequences using BLAT^64^ with parameters of “-minScore=20 - minIdentity=5 -maxIntron=10000 -tileSize=11”. The results were then parsed using MCC_splitter.pl which assigned each read to its corresponding bait target. The reads were then mapped to the genome using bowtie2^65^ with “-X 1000” parameter. The resulting SAM file was analyzed using MCC_analyser.pl.

### CIRCLE-seq.

Genomic DNA from HEK293T cells was isolated with a Gentra Puregene Kit (QIAGEN), used in accordance with the manufacturer’s instructions. CIRCLE-seq^38,66^ was performed as follows Purified genomic DNA was sheared with a Covaris S2 instrument to an average length of 300 bp. The fragmented DNA was end repaired, A-tailed, and ligated to an uracil-containing stem-loop adaptor, using the KAPA HTP Library Preparation Kit, PCR Free (KAPA Biosystems). Adaptor-ligated DNA was treated with Lambda Exonuclease (NEB) and *E. coli* Exonuclease I (NEB) and then with USER enzyme (NEB) and T4 polynucleotide kinase (NEB). Intramolecular circularization of the DNA was performed with T4 DNA ligase (NEB), and residual linear DNA was degraded by Plasmid-Safe ATP-dependent DNase (Lucigen). In vitro cleavage reactions were performed with 125 ng of Plasmid-Safe–treated circularized DNA, 90 nM Cas9-NG protein, Cas9 nuclease buffer (NEB), and 90 nM synthetic chemically modified sgRNA (−175) in a 50-μL volume. Cleaved products were A-tailed, ligated with a hairpin adaptor (NEB), treated with USER enzyme (NEB), and amplified by PCR with barcoded universal primers (NEBNext Multiplex Oligos for Illumina [NEB]), using Kapa HiFi Polymerase (KAPA Biosystems). Libraries were sequenced with 150-bp paired-end reads on an Illumina MiSeq instrument. CIRCLE-seq data analyses were performed using open-source CIRCLE-seq analysis software and default recommended parameters (https://github.com/tsailabSJ/circleseq). The parameters were: read_threshold: 4; window_size: 3; mapq_threshold: 50; start_threshold:1; gap_threshold: 3; mismatch_threshold: 6; search_radius: 30; merged_analysis: True. The CIRCLE-seq pipeline first merged the reads, which were then aligned to the genome using BWA^67^ mem. Off-targets were identified using findcleavagesites.py and were visualized using visualization.py.

### CasOFFinder.

Computational prediction of NG PAM-containing potential off-target sites with minimal mismatches relative to the intended target site (three or fewer mismatches overall, or two or fewer mismatches allowing rG:dU wobble base pairings to the protospacer sequence) was performed using CasOFFinder^68^.

### Targeted sequencing by rhAmpSeq.

On-target and off-target sites identified by CIRCLE-seq and CasOFFinder were amplified from genomic DNA from CD34+ donors by using the rhAMPSeq system (IDT). Sequencing libraries were generated according to the manufacturer’s instructions and sequenced with 151-bp paired-end reads on an Illumina NextSeq 550 instrument.

### Quantification of guide-dependent off-target editing activity.

The A to G editing frequency for each position on the protospacer sequence was quantified using CRISPRessoPooled (v2.0.41) with “–quantification_window_size 10 – quantification_window_center −10 –base_editor_output –conversion_nuc_from A – conversion_nuc_to G.” The genomic features of all off-targets identified computationally and experimentally were annotated using HOMER (v4.10)^69^.

### R-loop assay.

Triplicate cultures of HEK293T cell lines (ATCC, CRL#3216) were diluted to 1.25 × 10^5^ cells/mL in Dulbecco’s Modified Eagle’s Medium (DMEM) (Thermo Fisher, cat. no. 10569044) supplemented with 10% fetal bovine serum. The diluted HEK293T cells were then seeded in 48-well plates, using 14 wells for each of the three triplicate cell lines and a volume of 250 μL/well. At 24 h after plating, cells were lipofected with plasmids encoding nuclease-inactive SaCas9 (AddGene ID # 138162) and an SaCas9 sgRNA (spacer sequence: ATTTACAGCCTGGCCTTTGGGG; SaCas9 Site 2 from prior studies^41,44^). For each well, 300 ng of editor and 200 ng of guide plasmid were combined with 0.75 μL of Lipofectamine 2000 (Thermo Fisher Scientific) in 25 μL of Opti-MEM (Thermo Fisher Scientific). After the DNA–lipofectamine mixture was incubated for 5 min at room temperature, it was added to each well containing cells. At 24 h after lipofection, the culture medium was removed and the cells were resuspended by trypsinization with 40 μL of TrypLE Express (Thermo Fisher Scientific) per well. The cells were pooled, and the trypsin was quenched by adding a 2-fold excess volume of DMEM + 10% fetal bovine serum. The cells were centrifuged for 10 min at 300 rcf and washed in 10 mL of PBS. The cells were counted using a ChemoMetec NucleoCounter NC-3000. For each electroporation, 100,000 cells were separated into a new tube and centrifuged for 10 min at 300 × *g*. The PBS was removed, and the cells were resuspended in 5 μL of Lonza SF electroporation buffer per 100,000 cells. An aliquot of 5 μL of the cell suspension was placed in a 20-μL Lonza cuvette. Separately, editor mRNAs or RNPs were combined with 15 μL of Lonza SF buffer, then added to the cell suspensions in each cuvette and pipetted up and down once to mix the contents. To formulate editor RNPs, ABE7.10 or ABE8e protein was diluted to 5 μM or 8 μM, respectively, in 17 μL of SF buffer. Next, 15 μM or 28 μM of sgRNA targeting the −175 A>G edit was added and mixed by pipetting. RNPs were complexed for 20 min at room temperature before being added to cells. To formulate mRNA editors, 1.5 μL of editor mRNA (stock concentration: 2 μg/μL) and 0.5 μL of sgRNA (stock concentration: 100 μM) were added to 15 μL of SF buffer. Editor mRNAs were ordered as a custom product from TriLink BioTechnologies with a CleanCap AG 5’ cap modification, full substitution of uridine for N1-methylpseudouridine, and a 120-nucleotide polyA tail. After the cells and editors were combined, the cuvette contents were electroporated in a Lonza 4D nucleofector using program CM-130, and 80 μL of warmed DMEM +10% fetal bovine serum was added to each well directly after electroporation. After 10 min of recovery at 37 °C, 20 μL of the 100-μL cell solution was plated into one well of a 48-well plate containing 220 μL of pre-warmed DMEM + 10% fetal bovine serum. Three days after electroporation, the culture medium was removed and the cells were washed in 250 μL of PBS. After removal of the PBS, genomic DNA was extracted by adding 100 μL of PBS followed by freshly prepared lysis buffer (10 mM Tris-HCl, pH 8, 0.05% SDS, 25 μg/mL proteinase K (Thermo Fisher Scientific)). Lysis proceeded over 1 h at 37 °C and was followed by heat-inactivation of proteinase K at 80 °C for 30 min before PCR amplification of the on-target and off-target loci for high-throughput sequencing. PCR primers used are shown in Supplementary Table 14.

### Transcriptome-wide deamination analysis.

Healthy-donor CD34+ HSPCs were electroporated with ABE7.10 and ABE8e RNP targeting the −175 A>G site. Untreated and mock electroporated (EP ctrl) CD34+ cells were used as controls. Six days post electroporation, one million cells from each treatment condition were collected and the RNA was extracted using a Qiagen RNeasy Plus Mini kit (# 74136). Total RNA-seq was performed with 2–4 μg of RNA in an Illumina NovaSeq sequencer at 50 million reads per condition. FASTQ files were trimmed using trim_galore (version 0.6.4) with parameters of “–paired –clip_R1 6 –clip_R2 6”. The reads were mapped to the hg19 reference genome by using STAR (version 2.5.3a) with the following parameters: --twopassMode Basic –outSAMtype BAM SortedByCoordinate –outFilterMultimapNmax 20 – alignSJoverhangMin 8 –alignSJDBoverhangMin 1 –outFilterMismatchNmax 999 – outFilterMismatchNoverReadLmax 0.04 –alignIntronMin 20 –alignIntronMax 1000000 – alignMatesGapMax 1000000 –outSAMattributes Standard –sjdbScore 1. The resulting bam files were sorted and indexed using Picard (version 2.9.4). The average percentage of A-to-I editing was calculated using the method described in reference ^41^. Briefly, REDItools (v1.3) was used to quantify the percentage of A-to-I editing in each sample. All nucleotides except adenosines were then removed from the analysis, followed by all adenosines with a read q30-coverage of less than 10. Lastly, the number of adenosines that were converted to inosines in each sample was calculated and divided by the total number of adenosines^41^.

### Statistical analysis.

Data normality was checked using the Shapiro–Wilk test. The two-sample *t*-test or (exact) Wilcoxon rank sum test was used to compare two groups, where indicated in the figure legends. Linear regression analysis was also used to compare two groups adjusting for batch effects (Figs. 4b, 4c, 5b, 5h, 5k). The Fisher combination test was used to combine *P* values from exact Wilcoxon rank sum tests for each donor in order to compare ABE7.10 to UT to adjust for batch effects, and the exact Wilcoxon signed rank test was used for ABE8e (Fig. 5c). Linear regression analysis was used to model the relation between % on-target edits and %HbF, with adjustment for batch effects (Fig. 3c). A linear regression model was used to analyze data (Fig. 5d and 5i). The slope (coefficient estimate), the coefficient of multiple determination (R^2^), and the P value were calculated based on the linear model. The variation in the %HbF explained by the % on-target edits was calculated using the coefficient of multiple determination (R^2^) (Figs. 3c, 5d, 5i, Supplementary Table 4). The comparison of variances of %HbF across different batches within a single editing strategy was first performed using the Ansari–Bradley Test^70^ before the different batches of data were combined (Fig. 3, Extended Data Fig. 7, Supplementary Tables 3, 4, and 5). The comparison of SD in the %HbF in fully edited colonies across different editing strategies was first performed using the Ansari–Bradley Test^70^ by batch, then final *P* values across batches were combined using the Fisher combination test approach (Fig. 3d). The impact of allelic outcomes on %HbF for indel alleles with frequencies of ≥ 5% was assessed. The %HbF in edited colonies with the same indel was compared with that in untreated colonies by using same approaches as above. Linear regression analysis was also used to evaluate the association of specific Cas9 on-target γ-globin or *BCL11A* indels or the ABE7.10 edit with the %HbF in edited and untreated erythroid colonies. Adjustments for batch effects were made in the linear regression model for all γ-globin, −175, and BCL11A edited cells (Fig. 3e, f, and i, Extended Data Fig. 7, Supplementary Tables 4 and 5). The normality of the residual of the linear regression model was also checked using the Shapiro–Wilk test. The false discovery rate was controlled at 0.1 for screening mutations associated with the %HbF to correct for multiple comparisons. The selected mutations were associated with the %HbF in a multivariable linear regression model, adjusting for the batch effect by using stepwise model selection (Supplementary Table 5). All *P* values were two-sided.

## Extended Data

## Supplementary Material

Supplementary Information

Supplementary Tables

## Data Availability

The plasmid used for spCas9-NG, ABE8e-NG and ABE7.10-NG protein purification from bacteria has been deposited at AddGene (ID# 194705, 170663, 201190). DNA sequencing files can be accessed using the NCBI SRA. The CUT&RUN, RNA-seq, and Micro-Capture-C data are deposited in the Gene Expression Omnibus database (accession number GSE228822). All the sequencing data are mapped to the hg19 human genome. Source data are provided with this paper.
