# Supplementary material for: Potent and uniform fetal hemoglobin induction via base editing
Source: Nat Genet. Author manuscript; Available in PMC 2023 Dec 15. (PMC10722557; doi:10.1038/s41588-023-01434-7)
Supplement: Supplementary Information [file NIHMS1927139-supplement-Supplementary_Information.pdf]

Table of contents:

Supplementary Table 1 | Percentage of base editing or indels after ABE7.10 and Cas9 nuclease treatment in bulk CD34+ cells from healthy donors and %HbF.

Supplementary Table 2 | Linked to main Figure 3. Genotype and %HbF in erythroid colonies generated from ABE7.10 edited $CD34^+$ HSPCs.

Supplementary Table 3 | %HbF variance in erythroid colonies edited by Cas9 nuclease or ABE7.10.

Supplementary Table 4 | Associations of specific Cas9 nuclease indels or the ABE7.10 edit with HbF induction in erythroid colonies.

Supplementary Table 5 | Multivariate linear regression analysis showing the independent effects of specific indels in the γ-globin promoter BCL11A motif on %HbF in erythroid colonies.

Supplementary Table 6 | Linked to main Figure 4. Genotype and %HbF in erythroid colonies generated from ABE8e-edited $CD34^+$ HSPCs.

Supplementary Table 7 | Linked to main Figure 5. Percentage of base editing or indels after ABE7.10 or ABE8e editing of healthy donor $CD34^+$ cells pre- and post- xenotransplantation.

Supplementary Table 8 | Linked to main Figure 5. Percentage of base editing or indels after Cas9 nuclease or ABE7.10 or ABE8e editing of sickle cell disease donor $CD34^+$ cells pre- and post- xenotransplantation.

Supplementary Table 9 | Off-targets identified by CIRCLE-seq

Supplementary Table 10 | Cas-OFFinder predicted in silico off-targets.

Supplementary Table 11 | Off-target sites and primer sequences used for their amplification

Supplementary Table 12 | Sequences of single guide RNAs (sgRNAs) used in this study.

Supplementary Table 13 | Antibodies used in this study.

Supplementary Table 14 | Sequences of oligonucleotides used in this study.

Supplementary Table 15 | CRISpy parameters.

Supplementary Note 1 | Cas9-3xNLS expression & purification protocol

Supplementary Tables

Supplementary Table 1 | Linked to main Figure 1**.** Percentage of base editing or indels after ABE7.10 and Cas9 nuclease in bulk CD34+ cells from healthy donors and %HbF. Excel file.

Supplementary Table 2 | Linked to main Figure 3. Genotype and %HbF in erythroid colonies generated from ABE7.10-edited $CD34^+$ HSPCs. Excel file.

	HBG **(sd=19.2)**	***BCL11A*** **(sd=12.7)**	**-175 (sd=7.0)**
**UT (sd=10.6)**	0.0026	0.086	0.0074
HBG **(sd=19.2)**	1	0.23	1.6×10^{-5}
BCL11A **(sd=12.7)**	0.23	1	7.0×10^{-5}

Supplementary Table 3 | Linked to main Figure 3. %HbF variance in erythroid colonies edited by Cas9 nuclease or ABE7.10. Erythroid colonies were generated from $CD34^+$ cells edited with Cas9 nuclease or ABE7.10 and analyzed, as described in main Fig. 3. The table shows the standard deviation of %HbF in colonies with >90% target gene editing (shaded regions in main Figure 3a-c) and q-values for comparing variances between two editing strategies. *HBG*, Cas9 nuclease disruption of *BCL11A* motif in the γ-globin promoter (*HBG1* and *HBG2*); *BCL11A*, Cas9 nuclease disruption of the *BCL11A* intron 2 erythroid enhancer; -175, ABE7.10 installation of the γ-globin -175 T>C HPFH variant. UT, untreated control cells. sd, standard deviation. P-values determined by the Fisher's combination test on the p-values by four batches for comparing variance in %HbF between two different editing approaches calculated by using Ansari-Bradley test are shown. q-value was calculated to control false discovery rate using the Benjamini and Hochberg approach[1].

	Indel	EST	SE	P	R^2	q
HBG **n=200**	**-13**	0.29	0.028	2.6E-23	0.25	2.00E-22
	**-1**	-0.06	0.059	0.3	0.003	0.45
	**-3**	0.14	0.064	0.025	0.014	0.05
	**-2**	0.015	0.049	0.76	0.0003	0.83
	**-4**	0.26	0.068	0.00018	0.04	5.00E-04
	**+1**	-0.016	0.074	0.83	0.0001	0.83
BCL11A **n=123**	**-2**	0.2	0.079	0.013	0.03	0.017
	**-15**	0.23	0.074	0.0021	0.046	0.0042
	**-13**	0.19	0.083	0.022	0.03	0.022
	**+1**	0.33	0.034	1.30E-18	0.31	5.20E-18
**-175 n=181**	**TCT**	0.70	0.02	6.0E-82	0.81	NA

Supplementary Table 4 | Linked to Extended Data Figure 7. Associations of individual high-frequency indels of specific Cas9 nuclease- indels or the ABE7.10 edit with HbF induction in erythroid colonies. Indels are indicated for γ-globin (*HBG*) or *BCL11A* targeted with Cas9 nuclease. The -175T>C variant generated by ABE7.10 is indicated in the bottom row. 153, 105 and 40 untreated colonies were included in each analysis of *HBG*, *BCL11A* and -175 edited, respectively. EST, coefficient estimate. Positive values indicate association with HbF induction (increased %HbF); SE, Standard error; P, p-value determined by the t-test from the linear regression model; R^2, coefficient of multiple determination measuring the variation of %HbF resulting from individual indels; q, false discovery rate adjusted P-value calculated using the Benjamini and Hochberg approach[1]. EST and SE were calculated from linear regression model. We adjusted for the batch effect in the linear regression model for all *HBG*, *BCL11A*, and -175 edited colonies. NA is not available.

1. Benjamini, Y. & Hochberg, Y. Controlling the false discovery rate—a practical and powerful approach to multiple testing. *Journal of the Royal Statistical Society Series B-Statistical Methodology* **57**, 289-300 (1995).

Indel	EST	SE	P
**-13**	0.340	0.026	1.4E-31
**-4**	0.339	0.056	4.1E-09
**-3**	0.213	0.052	6.2E-05
**-2**	0.089	0.040	0.027

Supplementary Table 5 | Linked to main Figure 3. Multivariable linear regression analysis showing the independent effects of specific indels in the γ-globin (*HBG*) on %HbF in erythroid colonies. EST, coefficient estimate. SE, Standard error; P, P-value determined by the t-test from the linear regression model. Batch effect was adjusted for in the model.

Supplementary Table 6 | Linked to main Figure 4. Genotype and %HbF in erythroid colonies generated from ABE8e-edited $CD34^+$ HSPCs. Excel file.

Supplementary Table 7 | Linked to main Figure 5. Percentage of base editing or indels after ABE7.10 or ABE8e editing of healthy donor $CD34^+$ cells pre- and post- xenotransplantation. Editing was quantified by next generation sequencing at 3 days after editing (input) and in specific hematopoietic lineages 16 weeks after xenotransplantation. Excel file.

Supplementary Table 8 | Linked to main Figure 5. Percentage of base editing or indels after Cas nuclease or ABE7.10 or ABE8e editing of sickle cell disease donor $CD34^+$ cells pre- and post-xenotransplantation. Editing was quantified by next generation sequencing at 3 days after editing (input) and in specific hematopoietic lineages 16 weeks after xenotransplantation. Excel file.

Supplementary Table 9 | CIRCLE-seq identified off-targets. Excel file.

Supplementary Table 10 | Cas-OFFinder predicted off-targets. Excel file.

Supplementary Table 11 | Multiplex-targeted DNA sequencing to analyze potential off-target edits by ABE7.10 and ABE8e + γ-globin -175 sgRNA in healthy donor $CD34^+$ cells. Excel file.

Supplementary Table 12 | Sequences of single guide RNAs (sgRNAs) used in this study.

Name	Sequence (5′→3′)	PAM	Source
sgRNA -198	GTGGGGAAGGGGCCCCCAA	NGG	Synthego
sgRNA -175	AGATATTTGCATTGAGATAG	NG	Synthego/Biospring
sgRNA -113	CTTGACCAATAGCCTTGACA	NGG	Synthego
sgRNA HBG	CTTGTCAAGGCTATTGGTCA	NGG	Synthego/Biospring
sgRNA BCL11A	CTAACAGTTGCTTTTATCAC	NGG	Synthego
sgRNA -187/189	ATTGAGATAGTGTGGGGAAG	NGG	Synthego
sgRNA -175/187/189	AGATATTTGCATTGAGGTGG	NG	Synthego
sgRNA non-targeting	GTAGGCGCGCCGCTCTCTAC	-	Synthego
sgRNA AAVS1	ACCCCACAGTGGGGCCACTA	NG/NGG	Synthego

Supplementary Table 13 | Antibodies used in this study.

**Erythropoiesis**					
**Antibody and fluorophore**	**Clone**	**Dilutions used**	**Vendor**	**Catalog #**	**Reference (PMID using this antibody)**
Anti-Human CD235a FITC	GA-R2 (HIR2)	1:100	BD Pharmingen™	559943	31698466
Hoechst 33342	Stock 10 mM = 2000×	1:1000	Millipore Sigma	B2261	31698466
Anti-Human CD49d PE	9F10	1:20	BioLegend	304304	31698466
Anti-Human Band3 APC		1:100	New York Blood Center	Gift from X. An	31698466
Anti-Human Fetal Hemoglobin APC	REA5333	1:20	Miltenyi Biotec	130-108-243	19056937

**Chimerism after human/NBSGW xenotransplantation**					
Anti-Mouse CD45 FITC/BV786	30-F11 (RUO)/30-F11 (RUO)	1:40	BD Pharmingen™/ BD Horizon™	561088/564225	11062533/31698466

Anti-Mouse TER-119/Erythroid Cells PerCP-Cy™5.5	TER-119	1:40	BD Pharmingen™	560512	31698466
Anti-Human CD45 BV605	HI30	1:20	BD Horizon™	564047	31698466
Anti-Human CD33 PE-Cy™7	P67.6	1:20	BD Biosciences	333946	31698466
Anti-Human CD3 APC-Cy™7	SK7 (Leu-4) (RUO)	1:20	BD Pharmingen™	557832	31698466
Anti-Human CD19 (Leu™-12) PE/FITC	4G7 (IVD)/HIB19(RUO)	1:20	BD Biosciences/BD Pharmingen™	349209/555412	31698466 / 7690791
Anti-Human CD34 Alexa Flour 700/PE	581 (RUO)/581 (RUO)	1:20	BD Pharmingen™/ BD Pharmingen™	561440/555822	7680152
Anti-Human CD235a APC	GA-R2 (HIR2) (RUO)	1:20	BD Pharmingen™	551336	31698466
DAPI			ThermoFisher	R37606	

**CUT and RUN**					
Anti-GATA1 antibody		1:100	abcam	ab11852	33512425
TAL1 antibody		1:100	Gene Tex	GTX116020	33512425
LDB1 antibody		1:100	Rockland	600-401-350	NA
LMO2 antibody		1:100	Gene Tex	GTX48597	NA

Supplementary Table 14 | Sequences of oligonucleotides used in this study.

Name	Sequence (5′→3′)	Source	Assay
hHBG.F	TGACTGAATCGGAACAAGGCAAAGG	Integrated DNA Technologies (IDT)	Next-generation sequencing primers (overhangs not shown)
hHBG.R	ATTCTTCATCCCTAGCCAGCCGC	Integrated DNA Technologies (IDT)	
hBCL11A.F	GATACAGGGCTGGCTCTATGC	Integrated DNA Technologies (IDT)	
hBCL11A.R	CAAGAGAGCCTTCCGAAAGAGG	Integrated DNA Technologies (IDT)	
hAAVS1.F	AGTCTTCTTCCTCCAACCCGGGCCC	Integrated DNA Technologies (IDT)	

hAAVS1.R	CCTGCCAAGCTCTCCCTCCCAGGAT	Integrated DNA Technologies (IDT)	
4.9-kb Fwd	ACGGATAAGTAGATATTGAGGTAAGC	Integrated DNA Technologies (IDT)	qPCR detection of 4.9-kb deletion alleles
4.9-kb Rev	GTCTCTTTCAGTTAGCAGTGG	Integrated DNA Technologies (IDT)	
TaqMan probe (FAM)	ACTGCGCTGAAACTGTGGTCTTTATGA	Integrated DNA Technologies (IDT)	
Wild-type Fwd	AGCCTTGCCTTGACCAATAGCCTTGACAA	Integrated DNA Technologies (IDT)	Electrophoretic Mobility Shift Assay
Wild-type Rev	TTGTCAAGGCTATTGGTCAAGGCAAGGCT	Integrated DNA Technologies (IDT)	
+1 Fwd	AGCCTTGCCTTGAACCAATAGCCTTGACAA	Integrated DNA Technologies (IDT)	
+1 Rev	TTGTCAAGGCTATTGGTTCAAGGCAAGGCT	Integrated DNA Technologies (IDT)	
−1 Fwd	AGCCTTGCCTTGACAATAGCCTTGACAA	Integrated DNA Technologies (IDT)	
−1 Rev	TTGTCAAGGCTATTGTCAAGGCAAGGCT	Integrated DNA Technologies (IDT)	
−2 Fwd	AGCCTTGCCTTGAAATAGCCTTGACAA	Integrated DNA Technologies (IDT)	
−2 Rev	TTGTCAAGGCTATTTCAAGGCAAGGCT	Integrated DNA Technologies (IDT)	
−3 Fwd	AGCCTTGCCTTGAATAGCCTTGACAA	Integrated DNA Technologies (IDT)	
−3 Rev	TTGTCAAGGCTATTCAAGGCAAGGCT	Integrated DNA Technologies (IDT)	
HBG1/2 probe	CCTTGTCAAGGCTATTGGTCAAGGCAAGGCTGGCCAACCCATGGGTGGAGTTTAGCCAGGGACCGTTTCAGACAGATATTTGCATTGAGATAGTGTGGGGAAGGGGCCCCCAAGAGGATA	Integrated DNA Technologies (IDT)	Biotinylated probes used for Micro Capture-C
HS-3 probe-1	GCACTTGCCCCTAGCTGGGGGTATAGGGGAGCAGTCCCATGTAGTAGTAGAATGAAAAATGCTGCTATGCTGTGC	Integrated DNA Technologies (IDT)	
HS-3 probe-2	AGTCAAAATTCCTTGAAATCCAAGTCCTTAGAGACTCCTGCTCCCAAATTTACAGTCATAGACTTCTTCATGGCTGTCTC	Integrated DNA Technologies (IDT)	
Target_Site_Forward_Primer	ACACTCTTTCCCTACACGACGCTCTTCCGATCTNNNNCCTGGCCTCACTGGATACTC	Integrated DNA Technologies (IDT)	R-loop assay sequencing primers

Target_Site_Reverse_Primer	TGGAGTTCAGACGTGTGCTCTTCCGATCT CTGACAAAAGAAGTCCTGGTATC	Integrated DNA Technologies (IDT)	
Off-Target_Site_Forward_Primer	ACACTCTTTCCCTACACGACGCTCTTCCGATCTNNNNGGACATTTCCACCGCAAAATG	Integrated DNA Technologies (IDT)	
Off-Target_Site_Reverse_Primer	TGGAGTTCAGACGTGTGCTCTTCCGATGCTACAGAAAGGTCAGCAGC	Integrated DNA Technologies (IDT)	

Supplementary Note 1 | Cas9-3xNLS expression & purification protocol

1. Transform plasmid into BL21(DE3) to Kan resistance; plate o/n
2. Pick colony to Terrific Broth (TB) media with 25 ug/ml Kanamycin. Grow overnight at 37 °C
3. Inoculate into fresh TB Kan at 1:100 dilution. Grow at 37 °C to an OD600nm of 0.8
4. Reduce temperature to 20C. Induce with 0.5 mM IPTG for 20 h
5. Harvest cells
6. Resuspend cells in Lysis Buffer [25 mM Tris, pH 7.8, 500 mM NaCl, 10% glycerol, 1 mM TCEP] and lyse by 2 passages through microfluidizer.
7. Centrifuge lysate at 38,400 rcf for 1 h.
8. Transfer supernatant to fresh container and add Ni-NTA resin (Gold Biotech) [use 2ml beads per 1 L of culture]. Incubate at 4 °C for 1 h then apply to a BioRad gravity column.
9. Wash with 20 column volumes of 20 mM imidazole in lysis buffer.
10. Elute protein with 200 mM imidazole in lysis buffer
11. Analyze fractions by SDS-PAGE and pool fractions containing Cas9
12. Concentrate approximately 10-fold and estimate protein by A280 (Nanodrop).
13. Add 1 mg TEV (produced in-house) per 40 mg of Cas9. Incubate at 4 °C overnight.
14. Equilibrate a 5 ml HiTrap SP HP (Cytiva) with Buffer A [25 mM Tris, pH 7.8, 10% glycerol, 1 mM TCEP]
15. Dilute TEV-digested Cas9 sample to <100 mM NaCl with Buffer A and apply to SP column
16. Elute with a 0-80% gradient of Buffer B [25 mM Tris, pH 7.8, 1 M NaCl, 10% glycerol, 1 mM TCEP] over 25 column volumes. 5ml/min elution, 5 ml/tube
17. Analyze fractions by SDS-PAGE and pool those containing Cas9
18. Apply to a HiLoad Superdex 200 26/600 size exclusion column equilibrated with 25 mM Tris, pH 7.8, 500 mM NaCl, 10% glycerol, 1 mM TCEP. Elute isostatically with the same buffer
19. Analyze fractions by SDS-PAGE and pool those containing Cas9.
20. Apply to Detoxi-Gel (Thermo-Fisher) to remove endotoxin. Determine concentration at A280
